# A systematic review of real-world diabetes prevention programs: learnings from the last 15 years

**DOI:** 10.1186/s13012-015-0354-6

**Published:** 2015-12-15

**Authors:** Zahra Aziz, Pilvikki Absetz, John Oldroyd, Nicolaas P. Pronk, Brian Oldenburg

**Affiliations:** 1 Melbourne School of Population and Global Health, University of Melbourne, Melbourne, 3010 Australia; 2 School of Health Sciences, University of Tampere, Tampere, FI-33014 Finland; 3 Collaborative Care Systems Finland, Helsinki, Finland; 4 Department of Epidemiology and Preventive Medicine, Monash University, Melbourne, 3004 Australia; 5 HealthPartners Institute for Education and Research, 8170 33rd Ave. S, Minneapolis, MN 55425 USA

**Keywords:** Implementation, Translational research, Diabetes prevention, Penetration, Implementation, Participation, Effectiveness (PIPE) impact metric, Systematic review, Resource allocation

## Abstract

**Background:**

The evidence base for the prevention of type 2 diabetes mellitus (T2DM) has progressed rapidly from efficacy trials to real-world translational studies and practical implementation trials over the last 15 years. However, evidence for the effective implementation and translation of diabetes programs and their population impact needs to be established in ways that are different from measuring program effectiveness. We report the findings of a systematic review that focuses on identifying the critical success factors for implementing diabetes prevention programs in real-world settings.

**Methods:**

A systematic review of programs aimed at diabetes prevention was undertaken in order to evaluate their outcomes using the penetration, implementation, participation, and effectiveness (PIPE) impact metric. A search for relevant articles was carried out using PubMed (March 2015) and Web of Science, MEDLINE, CENTRAL, and EMBASE. A quality coding system was developed and included studies were rated independently by three researchers.

**Results:**

Thirty eight studies were included in the review. Almost all (92 %) provided details on *participation*; however, only 18 % reported the coverage of their target population (*penetration*). *Program intensity or implementation*—as measured by frequency of contacts during first year and intervention duration—was identified in all of the reported studies, and 84 % of the studies also reported implementation fidelity; however, only 18 % of studies employed quality assurance measures to assess the extent to which the program was delivered as planned. Sixteen and 26 % of studies reported ‘highly’ or ‘moderately’ positive changes (*effectiveness*) respectively, based on weight loss. Six (16 %) studies reported ‘high’ diabetes risk reduction but ‘low’ to ‘moderate’ weight loss only.

**Conclusion:**

Our findings identify that program intensity plays a major role in weight loss outcomes. However, programs that have high uptake—both in terms of good coverage of invitees and their willingness to accept the invitation—can still have considerable impact in lowering diabetes risk in a population, even with a low intensity intervention that only leads to low or moderate weight loss. From a public health perspective, this is an important finding, especially for resource constrained settings. More use of the PIPE framework components will facilitate increased uptake of T2DM prevention programs around the world.

**Electronic supplementary material:**

The online version of this article (doi:10.1186/s13012-015-0354-6) contains supplementary material, which is available to authorized users.

## Background

Type 2 diabetes mellitus (T2DM) has emerged as a major public health challenge with an estimated 387 million people living with T2DM globally [1]. Efforts to prevent or delay the onset of diabetes are an urgent public health priority with health, social, and economic benefits. Several large randomized controlled trials (RCTs) from the US, Finland, China, and India have demonstrated that lifestyle interventions can be successful in reducing the incidence of T2DM from 29–58 % in high-risk populations [2–5], with generally good maintenance for up to 20 years [6, 7]. However, these trials have mainly focused on efficacy and effectiveness outcomes. It is now important that more emphasis is given to the implementation and transferability of diabetes prevention programs into real-world settings. ‘Implementation’ research focuses on understanding the processes, results, and factors affecting implementation under real-world conditions by answering questions such as ‘why and how interventions work in real-world settings’ [8]. ‘Transferability’ describes the process of applying the results of research in one situation to other similar situations’ [9]. ‘Real-world’ contexts are settings where health research findings are applied in practice. The latter include primary healthcare settings, work organizations, churches, and schools. Major transferability gaps still remain in translating diabetes prevention from research into practice [10].

Several reviews of diabetes prevention trials have been conducted to examine their effectiveness [11–23]. Some recent reviews have examined how implementation influences effectiveness, most particularly in relation to the US diabetes prevention program (US-DPP). One of these reviews [24] summarized lifestyle interventions based on the US-DPP curriculum and described how different curricula (as a measure of implementation) affected outcomes. They found that the less-intense version of the US-DPP core curriculum may influence long-term outcomes. Ali et al. [25] conducted a systematic review and meta-analysis of 28 translational studies based on the US-DPP and concluded that the utilization of non-medical personnel in delivering diabetes prevention interventions can lower the overall costs without compromising effectiveness. Johnson and colleagues [26] synthesized evidence from translational studies and assessed the impact of interventions delivered outside large randomized trials. The review included 17 translational studies from a range of settings and concluded that there is potential for less intensive interventions to have an impact on future progression to diabetes in at-risk individuals. However, these reviews included studies where subjects had either already progressed to diabetes [25, 26] or had short follow-up (e.g. less than 12 months [26]).

Another systematic review by Dunkley et al. [27] concluded that pragmatic diabetes prevention programs are effective and adherence to international guideline recommendations is significantly associated with a greater weight loss; however, the authors mainly focussed on the effectiveness of the selected translational studies in relation to the main outcome without giving consideration to the adaptability and scalability dimensions of translation.

A more recent systematic review [28] assessing combined diet and physical activity promotion programs of between 3 months and 6 years duration concluded that more intensive programs resulted in greater weight loss and lowered the risk of T2DM more than less intensive programs and this was also found to be cost-effective. Based on the accumulated evidence from this and other reviews, the US Community Preventive Services Task Force (CPSTF) has recently recommended combined diet and physical activity promotion programs to prevent T2DM [28]. Importantly, however, the above review examining the translation of diabetes prevention programs like US-DPP did not address issues specifically related to the long-term scalability and sustainability of such programs.

Another comprehensive review to evaluate the generalizability of diabetes prevention trials was conducted by Whittemore [29] using Glasgow’s [30] reach, efficacy, adoption, implementation, maintenance (RE-AIM) framework. This is a framework designed to summarize how well research trials report on elements related to research translation. Generalizability is an important element of translational research, i.e. the degree to which findings from a study or set of studies can be more broadly generalized to populations and settings beyond those in the original studies [31, 32]. This review included 16 studies that translated the US-DPP protocol into four distinct settings: (a) hospital outpatient, (b) primary care, (c) community, and (d) work and church. The author found positive outcomes in terms of the reach, efficacy, adoption, implementation, and maintenance of programs. However, this review had a narrow focus and only included those studies that were based on the US-DPP; no other protocols were considered or included. Another limitation was that several studies considered only short-term effectiveness of up to 3 months, and some of the included studies also recruited patients who already had diabetes. Laws and colleagues [31] conducted a systematic review examining the adequacy of reporting of external validity components of lifestyle intervention trials aimed at T2DM prevention. The authors assessed the generalizability of the findings of 31 studies. Laws reported that all studies lacked full reporting on external validity elements. One of the limitations of this review was the use of a dichotomous rating scale (‘reported’ or ‘not reported’) which did not take into account external validity elements which were reported as continuous measures [31].

In summary, a number of recent reviews have focused on the real-world effectiveness of T2DM prevention, in particular, programs derived from the US-DPP. However, these reviews have not systematically examined the adaptability and scalability dimensions of translation, that is, what works, under what ‘real-world’ conditions and in which contexts; yet this information is critically important for policy makers and program implementers who need to identify diabetes prevention programs with significant population impact.

To address this knowledge gap, we have undertaken a systematic review that focuses on identifying the critical success factors for implementing diabetes prevention programs in real-world settings. We consider the importance of program and product design elements that are important to real-world implementation including the effect size, scope of services, scalability potential, and long-term sustainability [33]. We use the penetration, implementation, participation, effectiveness (PIPE) impact metric, a framework that is highly relevant to implementation and a formal assessment of the net impact of health improvement programs that is explicitly linked to the program design elements noted above [33]. The four key elements of the PIPE framework are as follows: (1) penetration of the program into the population of interest, (2) implementation of the proposed set of services, (3) participation in the program, and (4) effectiveness in generating expected outcomes. Each of the PIPE Impact Metric elements may be expressed as a coefficient, and the product of these four coefficients is referred to as the PIPE impact metric. The PIPE Impact Metric can be used to provide feedback to program administrators on gaps in performance, and it also enables the integration of program design features and identifies where to focus on for performance improvement changes. This paper aims to review the current evidence about success factors for implementing diabetes prevention programs in real-world settings using the PIPE Impact Metric.

## Methods

### Data sources and searches

A comprehensive search was carried out using PubMed, Web of Science, MEDLINE, CENTRAL, and EMBASE (February 2014). Search terms were ‘diabetes’ AND ‘prevention’ AND (‘program’ OR ‘intervention’) AND (implementation’ OR ‘translation’). The search was repeated using PubMed to include relevant articles from February 2014 to March 2015. A detailed search strategy is provided in Additional file 1.

### Study selection

We included all published studies in the last 15 years (i.e. 2001–2015) that reported on the evaluation of a lifestyle-focused program aimed at individuals at moderate or high risk of diabetes (e.g. impaired glucose tolerance (IGT), elevated haemoglobin A1c (HbA1c), high body mass index (BMI) or overweight). The inclusion criteria were adults aged 18 years or older; English language publications; and full text. The exclusion criteria were studies that were published prior to 2001; that did not report at least 1-year follow-up; included participants with known diabetes; included participants not at elevated risk of developing diabetes; and reported on multiple intervention components in a single study. Studies were also excluded if they were exclusively ‘diet-based’ or ‘exercise-based’ instead of referring to lifestyle or behavioural interventions or if they used lifestyle *and* pharmacological interventions.

### Data extraction

An evaluation of T2DM prevention program impact was conducted using the PIPE Impact Metric [33]. The PIPE Impact Metric expresses four elements of program initiation and long-term delivery needed to maximize the population impact (i.e., penetration, implementation, participation, and effectiveness) as a coefficient. The product of all coefficients becomes the PIPE Impact Metric. For example, a study may report having reached 250 out of 350 individuals at high risk for T2DM workers at a company. In this example, penetration is reported as (250/350) = 0.714 (71 %). Similar coefficients are calculated for implementation, participation, and effectiveness. As numeric data was not available for some of the elements in some of the included studies, an alternative coding system, also informed by PIPE, was developed for this paper.

### Data synthesis and analysis

The coding system is summarized in Table 1. It included two steps: (1) an initial scoring and (2) coding of the scores into ‘high’, ‘medium’, ‘low’, or ‘not able to calculate (NAC)’ where relevant data was not available. In the initial scoring, coefficients were calculated for penetration and participation. For implementation, the initial scoring was done on three aspects: frequency over the first 12 months; duration of the entire intervention including follow-up contacts; and intervention fidelity. To overcome heterogeneity in the kinds of contacts when scoring frequency, a system was developed to standardize the degree of contact based on number, length, and type (Table 1). Similarly, for effectiveness, the initial scoring was on three criteria: proportion of participants successful in achieving the main outcome; weight loss (in kilogrammes); and diabetes risk reduction (absolute/relative). The coding of the scores (into high, medium, low, and NAC) for each element was agreed-upon by the review team, and ratings were independently derived by three researchers (ZA, PA, JO). The ratings of all studies were then reviewed by all authors, and when disagreement on rating occurred, they were resolved with consensus among all authors.

**Table 1:** Development of coding system using PIPE Impact Metric elements

PIPE Element	Description	Coding of the scores
Penetration	Numerator: the number of individuals reached (invited)	≤33 % = low; 34–66 % = moderate; ≥67 % = high; or NAC (not able to calculate)
	Denominator: the number of individuals in target population	
Implementation	Implementation was rated on 3 aspects.	≤33 % = low; 34–66 % = moderate; or ≥67 % = high
	1. Frequency: the degree of contact (based on number, length, and type) over the first 12 months of an intervention. Different types of contacts were quantified based on the session type in the following way: • 1 group/individual session = 1 session • 1 group/individual session (>3 h) = 2 sessions • 1 online/telephone session = 0.5 session • 1 text/email/fax contact = 0.25 session	
	Numerator: total number of sessions (over the first 12 months)	
	Denominator: 22 (the US-DPP 16 weekly + 6 monthly = 22 sessions)	
	2. Duration: the duration of the intervention	≤6 months = low; 6–12 months = moderate; and >12 months = high
	3. Fidelity: the use of standard curriculum (for example: the US-DPP) for the delivery of intervention and use of quality assurance measures to monitor the implementation of the intervention	No standard curriculum followed = low; a standard curriculum was followed but no quality assurance measures were reported = moderate; a standard curriculum was followed and quality assurance measures were applied = high; or NAC (not able to calculate)
Participation	Numerator: the number of participants enrolled in the intervention	≤33 % = low; 34–66 % = moderate; ≥67 % = high; or NAC (not able to calculate)
	Denominator: the number of individuals reached (invited)	
Effectiveness	Effectiveness was rated on 3 criteria:	≤25 % = low; 26–40 % = moderate; >40 % = high; or NAC (where information is not provided)
	1. Success criterion/proportion of successful participants:	
	Numerator: participants who achieved the main outcome (i.e. weight loss ≥5 %)	
	Denominator: total number of participants enrolled in the intervention/total number of participants completed 12-month measurements	
	2. Average weight loss: the average weight loss (in kilogrammes)	≤2.3 kg = low; 2.4–4.6 kg = moderate; >4.6 kg = high; or NAC (where information is not provided)
	3. Risk reduction: diabetes risk reduction (absolute/relative)	Risk reduction:
		≤15 % = low; 16–30 % = moderate; >30 % = high; or NAC (where information is not provided)

## Results

**Fig. 1:**
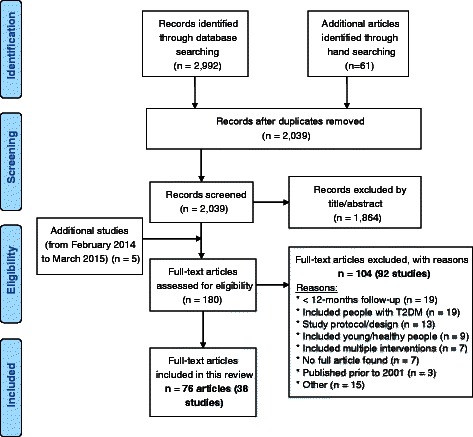
PRISMA flow diagram

A detailed PRISMA flow diagram is attached (Fig. 1). The initial literature search (February 2014) returned 2992 publications and 61 additional articles were identified through hand searching of references from the bibliographies of articles identified. Two thousand thirty-nine articles were screened after removing duplicates. An additional 5 articles were included after updating the search until March 2015. A total of 180 articles were assessed for eligibility. A total of 76 articles from 38 studies were included in the review. Table 2 describes the characteristics of the included studies.

**Table 2:** Characteristics of the included studies

Year	Author	Study ID	Country	Setting	Study population	Sample size	Study design	Intervention
2003	Mensink et al. [35]	SLIM	Netherlands	Unclear	Adults at risk of T2DM	114	RCT	3 individual and 1 group session during 1 year + participants were encouraged to participate in the exercise program 3 times a year
2005	Kosaka et al. [62]	Japanese DPP	Japan	Hospital-based	Adults with IGT	458	RCT	Detailed instructions on lifestyle were repeated every 3 to 4 months during hospital visits
2006	Oldroyd et al. [63]	Newcastle lifestyle intervention	UK	Primary care	Adults with IGT	78	RCT	12 individual 15- to 20-min review appointments over 24 months (6 in the first 6 months, 1 after 9 months and 5 at 2 monthly intervals between 12 and 24 months)
2007	Absetz et al. [41]	GOAL LIT	Finland	Primary care	Adults at risk of T2DM	352	Before and after	Six 2-hourly group counselling sessions over 8 months
2007	Bo et al. [36]	Italian Trial	Italy	Primary care	Adults with metabolic syndrome	335	RCT	1 individual and four 1-hourly group sessions
2007	Davis-Smith et al. [39]	DPP (church-based)	USA	Community (church)	Adults at risk of T2DM	10	Before and after	6-session lifestyle intervention delivered over a 7 week period
2007	Laatikainen et al. [64]	Greater Green Triangle (GGT)	Australia	Primary care	Adults at risk of T2DM	311	Before and after	6 structured 90-min group sessions delivered during an 8-month period
2008	Ackermann et al. [65]	DEPLOY	USA	Community (YMCA)	Adults at risk of T2DM	92	RCT	Sixteen 1 to 1.5-hourly small group sessions over 16 to 20 weeks and monthly large-group meetings
2008	Boltri et al. [66]	DPP (church-based)	USA	Community (church)	Adults with pre-T2DM	8	Before and after	16 weekly group sessions conducted over 4 months
2008	Payne et al. [67]	BDPPI	Australia	Outpatient setting	Adults at risk of T2DM	122	Before and after	6-week group self-management education program, 12-week gym- or home-based resistance training, and three 2-h group reinforcement sessions during 34-week maintenance program
2009	Kramer et al. [68]	GLB (2007–2009)	USA	Community	Adults with pre-T2DM	42	Before and after	12 weekly sessions (~60 min) and participants were offered the opportunity to attend monthly support meetings for 9 months after completion of the intervention
2009	Kulzer et al. [69]	PREDIAS	Germany	Outpatient setting	Adults at risk of T2DM	182	RCT	12 lessons lasting ~90 min each
2009	Penn et al. [70]	EDIPS—Newcastle	UK	Outpatient setting	Adults with IGT	102	RCT	A 30-min session immediately following randomisation and 2 weeks later, then monthly for the first 3 months and every 3 months thereafter up to 5 years
2010	Almeida et al. [71]	Colorado weight loss intervention	USA	Integrated health care organization	Adults with pre- T2DM	1520	Matched cohort	A single 90-min small group session
2010	Makrilakis et al. [72]	DE-PLAN Greece	Greece	Primary care (workplace)	Adults at risk of T2DM	191	Before and after	6 sessions (1 h each) held by a registered dietician in the groups of 6 to 10 persons
2010	Parikh et al. [73]	Project HEED	USA	Community	Adults at risk of T2DM	99	RCT	A peer-led lifestyle intervention group, presented in a workshop consisting of eight 1.5-h sessions over 10 weeks
2010	Vanderwood et al. [45]	Montana CVD and DPP	USA	Health care facilities	Adults at risk of T2DM and CVD	355	Before and after (pilot study)	16 weekly group sessions and 6 monthly group sessions
2010	Vermunt et al. [74]	APHRODITE	Netherlands	Primary care	Adults at risk of T2DM	925	RCT	11 consultations of 20-min over 2.5 years, five 1-h group meetings and 1-h personal consultation with the dietician
2011	Boltri et al. [75]	DPP (church-based)	USA	Community (church)	Adults with pre-T2DM	37	Before and after	6 or 16 weekly group sessions
2011	Gilis-Januszewska et al. [76]	DE-PLAN Poland	Poland	Primary care	Adults at risk of T2DM	175	Prospective cohort	10 group sessions lasting for 4 months, 6 telephone motivation sessions, and 2 motivation letters sent to participant
2011	Katula et al. [77]	HELP PD	USA	Community (various venues)	Adults with pre-T2DM	301	RCT	~26 weekly group sessions for the first 6 months, 3 personalized consultations with a registered dietician, 18 monthly group sessions, and monthly phone contact for the last 18 months
2011	Kumanyika et al. [48]	Think health!	USA	Primary care	Adults with high BMI and weight	261	RCT	Brief monthly contact with a lifestyle coach (LC) for 12 months and 10–15 min counselling sessions with primary care providers every 4 months. Bi-monthly sessions with LC for the second year
2011	Nilsen et al. [42]	Nilsen et al.	Norway	Primary care	Adults at risk of T2DM	213	RCT	The individual and interdisciplinary group participated in a group-based program, 1 day (5 h per day) each week for 6 weeks
								An individual 30-min consultation with a nurse or ergonomist completed the intervention 1 month after the last group meeting
2011	Penn et al. [43]	NLNY	UK	Leisure and community settings	Adults at risk of T2DM	218	Before and after	A 10-week program of twice-weekly 1.5-h sessions, followed by ongoing support with regular mobile phone text message and email reminders, ‘drop-in’ activity sessions continued up to 12 months
2011	Ruggiero et al. [78]	HLP	USA	Community (various venues)	Adults at risk of T2DM	69	Before and after	16 weekly core sessions and 6 monthly after-core sessions
2011	Sakane et al. [79]	Japanese Study	Japan	Primary care (workplace)	Adults with IGT	304	RCT	4 group sessions of 2 to 3 h (for the first 6 months), individual sessions twice a year for 3 years. Between-visit contact by fax was also made monthly during the initial 12 months
2012	Costa et al. [40]	DE-PLAN-CAT	Spain	Primary care	Adults at risk of T2DM	552	Prospective cohort	A 6-h educational program (scheduled in 2 to 4 individual/small group sessions), and regular contact by phone or text message for at least once every 6 to 8 weeks
2012	Janus et al. [46]	pMDPS	Australia	Primary care	Adults at risk of T2DM	92	RCT	6 structured 90-min group sessions. The first 5 sessions were at 2 weeks intervals and the final session was 8 months after the first
2012	Kanaya et al. [50]	LWBW	USA	Community	Adults at risk of T2DM	238	RCT	The intervention was primarily telephone-based counselling (12 calls) with 2 in-person sessions and 5 optional group workshops over 1 year period
2012	Lakerveld et al. [37]	Hoorn Prevention Study	Netherlands	Primary care	Adults at risk of T2DM and/or CVD	622	RCT	Six individual 30-min counselling sessions, followed by 3-monthly booster sessions by phone for a period of 1 year.
2012	Ockene et al. [80]	LLDPP	USA	Community	Adults at risk of T2DM	312	RCT	3 individual and 13 group sessions over a 12 month period
2012	Piatt et al. [81]	GLB (2005–2008)	USA	Community	Adults with metabolic syndrome	105	Before and after	12 weekly sessions over 12 to 14 weeks (lasted ~90 min) in the groups of 5 to 13 participants
2013	Jiang et al. [82]	SDPI-DP	USA	Community	Adults with pre-T2DM	2553	Before and after	16 group sessions in the first 16 to 24 weeks and monthly individual lifestyle coaching sessions
2013	Ma J et al. [38]	E-LITE	USA	Primary care	Overweight/obese adults with increased cardiometabolic risk	241	RCT	12 weekly group sessions (1.5 to 2 h each) in the first 3 months. From month 4 to 15, contact every 2 to 4 weeks depending on participant needs and preferences. Individual, secure email/phone contacts with personalized progress feedback and lifestyle coaching throughout the maintenance phase (month 4 to 15)
2014	Duijzer et al. [49]	SLIMMER	Netherlands	Primary care	Adults at risk of T2DM	31	One group pre-test post-test	In addition to 6 individual consultations (in total 4 h per participant), on average, participants received 5.2 consultations by dieticians and 34.1 sports lessons
2014	Sepah et al. [47]	Prevent	USA	Online platform	Adults with pre-T2DM	220	Quasi-experimental research design	16 online weekly lessons. Participants were then offered to continue with a post-core lifestyle change maintenance intervention, with the entire intervention (core plus post-core) totalling 12 months
2014	Zyriax et al. [34]	DELIGHT	Germany	Primary care (workplace)	Adults at risk of T2DM	241	Before and after	12 weekly sessions (for the first 6 months), 6 monthly and 6 biweekly sessions (for the next 6 months). For year 2 and 3 quarterly 1.5-h sessions
2015	Savas et al. [44]	IGT care call	UK	Primary care	Individuals with IGT	55	Observational study	A telephone service providing a 6 month lifestyle education program (20 min × 6), in addition to an introduction call (10 min) and action planning call (40 min)

Studies that met the eligibility criteria for this review were mainly based on either the US-DPP or Finnish-DPS. Seventeen (45 %) studies were implemented in the USA, 4 in the UK, 4 in the Netherlands, 8 in other European countries, 3 in Australia, and 2 in Japan. There were no studies from low and middle income countries that met our eligibility criteria. The sample size for the participants enrolled in each of these studies ranged between 8 and 2553 participants. The studies were conducted in a range of settings including primary health care, faith-based, workplace, and other community-based settings.

Each study was assessed on the four components of PIPE Impact Metric using a coding system described in Table 1. Table 3 describes the ratings of all included studies based on the elements of the PIPE Impact Metric.

**Table 3:** Scoring for each PIPE element by study

Author Year	Study	Penetration	Implementation			Participation	Effectiveness		
			Frequency	Duration	Fidelity		Success rate	Weight loss	Risk reduction (absolute/relative)
Mensink et al. 2003 [35]	SLIM	High	Low	High	Low	Low	NAC	Moderate	High
Kosaka et al. 2005 [62]	Japanese DPP	NAC	Low	High	Moderate	NAC	NAC	Moderate	High
Oldroyd et al. 2006 [63]	Newcastle LI	NAC	Moderate	High	NAC	Low	NAC	Low	NR
Absetz et al. 2007 [41]	GOAL LIT	NAC	Moderate	Moderate	Moderate	High	NAC	Low	NR
Bo et al. 2007 [36]	Italian Trial	High	Low	High	High	Low	NAC	Low	High
Davis-Smith et al. 2007 [39]	DPP (church-based)	Moderate	Low	Low	Moderate	Low	NAC	High	NR
Laatikainen et al. 2007 [64]	GGT	NAC	Low	Moderate	High	Low	NAC	Moderate	NR
Ackermann et al. 2008 [65]	DEPLOY	NAC	High	High	Moderate	Low	NAC	High	NR
Boltri et al. 2008 [66]	DPP (church-based)	NAC	High	Low	Moderate	Low	NAC	Low	NR
Payne et al. 2008 [67]	BDPPI	NAC	High	High	Moderate	NAC	Moderate	Moderate	NR
Kramer et al. 2009 [68]	GLB (2007 – 2009)	NAC	High	Moderate	Moderate	Low	NAC	High	NR
Kulzer et al. 2009 [69]	PREDIAS	NAC	Moderate	Low	Moderate	NAC	NAC	Moderate	NR
Penn et al. 2009 [70]	EDIPS- Newcastle	NAC	Moderate	High	NAC	Low	NAC	Low	High
Almeida et al. 2010 [71]	Colorado weight loss intervention	NAC	Low	Low	NAC	Low	Low	Low	NR
Makrilakis et al. 2010 [72]	DE-PLAN Greece	NAC	Low	Moderate	Low	Low	NAC	Low	NR
Parikh et al. 2010 [73]	Project HEED	NAC	Moderate	Low	Moderate	Low	Moderate	Moderate	NR
Vanderwood et al. 2010 [45]	Montana CDDP	NAC	High	Moderate	Moderate	High	High	High	NR
Vermunt et al. 2010 [74]	APHRODITE	NAC	Moderate	High	NAC	Low	NAC	NAC	NR
Boltri et al. 2011 [75]	DPP (church-based)	NAC	Low (2 churches)	Low	Moderate	Low	NAC	Low	NR
			High (3 churches)						
Gilis-Januszewska et al. 2011 [76]	DE-PLAN Poland	NAC	Moderate	Moderate	Low	Low	Low	Low	NR
Katula et al. 2011 [77]	HELP PD	NAC	High	High	High	Low	NAC	High	NR
Kumanyika et al. 2011 [48]	Think Health!	NAC	Moderate	Moderate	Moderate	Moderate	Low	Low	NR
Nilsen et al. 2011 [42]	Nilsen et al.	NAC	High	High	NAC	High	Moderate	NAC	NR
Penn et al. 2011 [43]	NLNY	NAC	High	Moderate	Low	High	Low	Low	NR
Ruggiero et al. 2011 [78]	HLP	NAC	High	Moderate	Moderate	Low	Moderate	Low	NR
Sakane et al. 2011 [79]	Japanese Study	NAC	Moderate	High	Moderate	Low	NAC	Low	High
Costa et al. 2012 [40]	DE-PLAN-CAT	Low	Low	High	NAC	Low	NAC	Low	High
Janus et al. 2012 [46]	pMDPS	NAC	Low	Moderate	High	High	NAC	Moderate	Moderate
Kanaya et al. 2012 [50]	LWBW	NAC	Moderate	Moderate	Moderate	Moderate	NAC	Low	NR
Lakerveld et al. 2012 [37]	Hoorn Prevention Study	High	Moderate	Moderate	High	Low	NAC	NAC	NR
Ockene et al. 2012 [80]	LLDPP	NAC	High	Moderate	Moderate	Low	NAC	Low	NR
Piatt et al. 2012 [81]	GLB (2005–2008)	NAC	Moderate	Moderate	Moderate	Low	Moderate	NAC	NR
Jiang et al. 2013 [82]	SDPI-DP	NAC	High	Moderate	Moderate	Low	NAC	Moderate	NR
Ma J et al. 2013 [38]	E-LITE	High	High	High	High	Low	High	High	NR
Duijzer et al. 2014 [49]	SLIMMER	NAC	Low	Moderate	Moderate	Moderate	NAC	Moderate	NR
Sepah et al. 2014 [47]	Prevent	NAC	Moderate	High	Moderate	High	High	NAC	NR
Zyriax et al. 2014 [34]	DELIGHT	High	High	High	Low	Low	NAC	NAC	NR
Savas et al. 2015 [44]	IGT Care Call	NAC	Low	Moderate	High	High	NAC	Moderate	NR

Details on the scoring of all included studies based on the elements of the PIPE Impact Metric framework are provided in Additional file 2: Table S4–Table S7

*NAC* not able to calculate, *NR* not reported

### Program penetration

Our analysis shows that only nine (24 %) studies reported their estimated target population, from which only seven studies reported the proportion of the target population that was reached with invitations to engage in the program or intervention. Out of these seven studies, five had ‘high’, one had ‘moderate’, and one had ‘low’ penetration into their target populations (see Table 3 and Additional file 2: Table S4). Target populations included patients, employees, and church attendees. Each study used various strategies to recruit potential participants including mail invitations, posted flyers, advertising through media, contacting local physicians, local churches, or using intranet or work meetings in the workplace setting.

The five studies that were rated as having ‘high’ penetration in our analysis applied heterogeneous strategies to reach their target group. Two studies contacted a pre-defined group of people at risk: one at worksite, where all employees who had above average waist circumference were invited for screening [34], and in the other study [35] all eligible subjects with high risk for glucose intolerance from a cohort representing general population were contacted. Three studies contacted 100 % [36], 68 % [37], and 70 % [38] of the target population for selective screening either by mail or by appointment. The only study rated as having ‘moderate’ penetration, was a church-based study [39]. The church roster included 407 members, whereas 37 % of adults (approximately 150) who attended Sunday gatherings were invited to complete a diabetes risk assessment. The study rated as having ‘low’ penetration in our analysis [40] included 18 participating centres that covered all primary care services for 4.5 % of the population in Catalonia, out of which less than 1 % of the population was invited for screening.

### Program implementation

In order to assess implementation, we evaluated the degree of contact (based on number, length, and type) during the first year of the intervention as frequency; the duration of the entire intervention; and the fidelity of the intervention (see Table 1). All studies in our analysis reported on frequency and duration. Thirty-four percent of all studies implemented ‘high’ frequency interventions, and 39 % studies delivered intervention over the period of 12 months or more. The number of contacts varied from a single small group session to 32 group sessions. About two thirds (66 %) of the programs based on the US-DPP model adopted a ‘low-’ to ‘moderately intense’ version (based on the degree of contact) as compared to the original. The main adaptation was the reduction from 16 to fewer sessions. In addition, groups led by volunteers as opposed to health care professionals and use of telephone as opposed to face-to-face delivery of individual sessions were also frequently observed adaptations. Only a small proportion (16 %) of studies reported ‘low’ duration, i.e. intervention delivered over the period of 6 months or less.

Implementation fidelity was defined as intervention being based on a standard curriculum for example the US-DPP and whether any quality assurance measures were applied to monitor the implementation of the intervention. Twenty-seven (71 %) studies were based on a standard curriculum, out of which only 7 studies had quality assurance measures applied to monitor the implementation. In some of these studies, while authors reported on the efforts made to minimize the potential lack of fidelity, none of these studies provided information on the extent to which the various components were delivered except one where the program components were more frequently added (40 %) than omitted (28 %) [41]. Refer to Table 3 and Additional file 2: Table S5 for further details.

### Program participation

The majority of the studies in our analysis (*n* = 35; 92 %) reported participation. Twenty-five (71 %) of these 35 studies achieved ‘low’ participation rates. Half of these studies achieved participation rates equivalent to or lower than 10 %. Only 7 (18 %) studies had ‘high’ participation rates. For four of these studies [41–44], participants were recruited by referral from physicians, general practitioners (GPs), or nurses from the participating health facilities and invited to attend a screening clinic. Two of the 7 studies [45, 46] used a combination of strategies to recruit potential participants including contacting local physicians and primary healthcare practices; advertising through media; and recruiting through local employers, work sites, churches, and service groups. One study [47] recruited participants from online advertisement, seeking individuals with a self-reported clinical diagnosis of prediabetes occurring within the past year; however, recruitment was based on self-selection by participants, which does not reflect a truly random sample [47].

Three studies were scored as having ‘moderate’ participation, where 63 % [48], 57 % [49], and 44 % [50] of the individuals were enrolled after assessing all ‘invited’ individuals, for study eligibility (see Table 3 and Additional file 2: Table S6). In most of the remaining studies, the ‘low’ participation was attributed to either the non-eligibility of potential participants or the refusal to participate.

For studies where information was available for both penetration and participation, it was observed that ‘high’ penetration into the target population did not have positive effect on participation. All five studies rated as ‘high’ penetration in the analysis, reported ‘low’ participation. Also, none of the seven studies that were rated as ‘high’ participation provided enough information on reaching out to their target populations, and hence, penetration could not be calculated. However, the information available suggests that the studies where high-risk participants were identified and referred through their GPs or nurses resulted in ‘high’ participation rates.

### Program effectiveness

Effectiveness was rated based on three criteria: proportion of successful participants; average weight loss; and diabetes risk reduction (absolute/relative) (Refer to Table 1). None of the studies reported on all three criteria. Seventeen (45 %) studies reported the use of intent-to-treat analysis; however, for the purpose of our analysis, effectiveness indicators were considered as presented in each of the studies.

One third (*n* = 12, 32 %) of the studies reported the proportion of successful participants who achieved the primary outcome (i.e. 5 % weight loss). The proportion of successful participants ranged between 20 and 64 %. Thirty-two (84 %) studies reported average weight loss by participants at 12 months, with a range from 0.45 to 7.7 kg. Only six of these studies were rated ‘high’ where average weight loss by participants was more than 4.6 kg. Sixteen (42 %) studies were rated ‘low’ on the basis of average weight loss of ≤2.3 kg. Only seven (18 %) studies reported the data on diabetes risk reduction (absolute/relative), where six studies were rated ‘high’ and one was rated ‘moderate’. Scores could not be calculated in three studies due to the lack of numerical data.

Thirteen studies (34 %) were rated ‘high’ on at least one of the three criteria. One study [45] that was rated ‘high’ on both success rate and weight loss, reported that 64 % of participants achieved 5 % weight loss goal with the average 12-month weight loss of 7.7 kg. This study was not only ‘highly’ effective but also had ‘high’ participation rates. The intervention included ‘high’ number of sessions with ‘moderate’ duration and ‘moderate’ fidelity. Another study [38] that had ‘high’ penetration into its target population appeared to be ‘highly’ effective based on the proportion of successful participants (50 %) and average weight loss (6.3 kg at 15 months). This study however, reported ‘low’ participation and delivered ‘high’ number of sessions and had ‘high’ duration of intervention.

Five out of six studies that reported ‘high’ weight loss delivered ‘high’ number of sessions.

Out of 10 studies that reported ‘moderate’ weight loss, 6 (60 %) implemented interventions with ‘low’ number of sessions/contacts. Sixteen (42 %) studies had ‘low’ effectiveness based on average weight loss, out of these, 8 (50 %) studies delivered ‘moderate’ number of sessions, whereas 4 (25 %) delivered ‘high’ and 4 (25 %) delivered ‘low’ number of sessions. Six studies reported ‘high’ effectiveness in risk reduction despite ‘low’ (4 studies) or ‘moderate’ (2 studies) effectiveness in weight loss, and these were also all studies with only ‘low’ (4 studies) or ‘moderate’ (2 studies) frequency, but with ‘high’ duration (6 studies). See Table 3 and Additional file 2: Table S7 for more details.

## Discussion

This is the first systematic review to evaluate the implementation of real-world diabetes prevention programs using the PIPE Impact Metric framework that deploys four highly relevant elements for monitoring program impact in real-world settings. As such, this review complements other recent reviews, e.g. Dunkley et al. 2014 [27], by providing a more detailed understanding of key factors underlying successful translation and implementation of diabetes prevention programs in real-world contexts. We have also defined the specific scope of services for calculating the overall costs of services being provided. From both an organizational and societal perspectives, these issues are important to consider since the relative costs and benefits of such services and programs are important determinants of their uptake and adoption. Our review of studies published over the last 15 years aims to identify the components of diabetes prevention programs with the highest population impact.

Our review highlights several important findings. First, confirming earlier reviews, our analysis demonstrates that lifestyle-focused diabetes prevention programs that have a ‘high’ degree of contact have more potential to achieve effective outcomes, especially when measured by weight loss. These programs have typically been based on the US-DPP model and have used a very structured protocol to maximize program fidelity. However, the problem with this approach is that in these studies, program participation tends to be quite low; and furthermore, none of these studies reported diabetes risk reduction.

Second, six of the studies showed different degrees of effectiveness for different outcomes. For example, diabetes risk reduction could be ‘high’ even when effectiveness in weight loss was ‘low’ or ‘moderate’. Surprisingly, these were all studies of ‘low’ or ‘moderate’ frequency, but ‘high’ duration. This could be very promising especially for settings where intervention resources are constrained but when large populations can be reached by such programs.

Third, we found that ‘high’ penetration into the target population with invitations to engage prospective participants in the program do not necessarily result in ‘high’ participation. However, three studies with ‘high’ penetration resulted in either ‘high’ weight loss or ‘high’ diabetes risk reduction. Hence, scalability of the program to reach a large audience appears to be an important ingredient for population-level impact.

In summary, while an intensive intervention plays an important role in achieving successful weight loss outcomes, highly scalable moderate- to low-frequency interventions appear to have major potential to achieve diabetes risk reduction in populations.

From a program implementation perspective, it is important to clearly define and estimate the size of the target population. Without a reasonable estimate of the size of the target population, there is a risk that the program will not be scalable or sustainable [33]. We found estimates of target populations to be reported by less than one third of included studies. Hence, it is possible that many of these programs were delivered to highly selected populations which limit their generalizability.

We used the US-DPP intervention as a benchmark to assess the intensity of dose-delivered (including frequency, duration, and fidelity) in our included studies. In order to deal with heterogeneity in the types of contacts, we constructed a framework to standardize the degree of contact based on number, length, and type. We found that many programs adopted a ‘low-’ to ‘moderately intense’ version, compared to the US-DPP intervention. In practice, this also means that many of the components were either omitted or modified from the original US-DPP curriculum. As stated earlier, we also note that ‘high’-frequency interventions with ‘high’ to ‘moderate’ duration and fidelity were associated with greater weight loss. This is consistent with a systematic review and meta-analysis by Dunkley et al. [27], where reviewers coded intervention content based on the recommendations for lifestyle interventions for the prevention of diabetes provided by both the European Guideline and Training Standard for Diabetes Prevention (IMAGE) project [51] and National Institute for Health and Care Excellence (NICE) [52] and found that adherence to guidelines on the content and delivery was significantly associated with a greater weight loss. Our findings on the potential of ‘low’ to ‘moderate’ frequency interventions with longer duration to achieve significant risk reduction support earlier findings [25, 26]. A recent CPSTF review [28] shows lower weight loss than the US-DPP but still concluded strong evidence of effectiveness. The recommendations from this review were further supported by an effectiveness and economic review [53, 54]. So, in general, it seems that studies beyond the original US-DPP generate somewhat lower effect for weight loss but still generate meaningful positive impact on the reduction of incidence of T2DM. Lindstrom et al. have also previously discussed the positive effects of decreased fat and increased fibre intake on diabetes risk reduction in the absence of weight loss [55]. We also know that in many populations diabetes risk can be high at lower levels of weight, with other factors besides weight loss playing a critical role in risk reduction [56, 57].

Examining the implementation component further, in calculating ‘frequency’, we have used contacts made in the initial 12 months only because most of the studies did not extend beyond 12 months. In those that did, the initial 12 months can be considered as the ‘action’ phase, bringing about the lifestyle changes, and beyond that is a follow-up and maintenance phase, which some studies support with less frequent contacts.

In translational research, a systematic evaluation of program fidelity is important to assess the extent to which program was implemented as designed. We based our definition of fidelity on whether a standard curriculum was used to guide the delivery of intervention and whether quality assurance measures were placed to monitor the implementation of the intervention. Not many studies clearly reported whether the quality assurance measures were able to guarantee ‘high’ fidelity, this clearly being one of the next important steps in program development. This needs to be examined in future studies.

All but three studies in our review reported participation, and only seven studies scored ‘high’ on participation rates. The high participation may be a reflection of highly targeted penetration, but because of unavailability of information, we could not calculate penetration coefficients for any of these seven studies. However, the information available suggests that the studies where high-risk participants were identified and referred through their GPs or nurses resulted in ‘high’ participation rates, underlying the important role of providers. The high participation could also be a reflection of high motivation for change among at-risk individuals, a factor that the providers can further enhance in face-to-face contact, e.g. by applying strategies from motivational interviewing [58]. In our review, of the seven studies that scored ‘high’ for participation, five studies reported ‘high’ to ‘moderate’ effectiveness based on success rate and/or weight loss.

The PIPE Impact Metric elements are interrelated in that participation is always a proportion of penetration and effectiveness can only be attributed to those who participated. Effectiveness, in this context, is defined as the number or proportion of participating cases who reached a priori defined success criterion. In prevention of T2DM, diabetes risk reduction is one such success criterion. In many studies, however, weight loss was also a main outcome—either in individual cases as a percentage of overall body weight or across a population as an average percentage of weight loss. In our review, we used either criterion—diabetes risk reduction or weight loss—to assess effectiveness.

We found that weight loss was reported in several different ways. For example, the percentage weight lost could be interpreted to mean weight loss to percentage achieving a particular weight loss target or the average percentage of weight that subject lost. One study [45] reported both 5 and 7 % weight loss, whereas another [40] reported the proportion of successful participants who achieved at least 3 % weight loss. For consistency, in our review, we only used 5 % weight loss when it was reported in several different ways.

However, examination of studies with ‘low’ effectiveness reveals that for some of the studies the reported changes in weight loss were very small. Some of these studies reported a significant reduction in weight following the active intervention phase, but the weight was partly or, in some studies, entirely regained by the end of 12 months. Lack of consistency in the way weight loss outcomes are reported and analysed needs to be addressed in future translational research [29]. Cardona-Morrell and colleagues [21] suggests the establishment of a registry of translational projects using consistent, measurable outcomes to add more certainty to effectiveness analyses.

Our review includes diabetes prevention translational programs published since 2001 and until 2015. The studies included in this review have implemented ‘high’ (34 %), ‘moderate’ (37 %), and ‘low’ (29 %) frequency interventions; however, we noticed that most of the ‘low-frequency’ interventions were conducted in earlier years, whereas, designing ‘moderate’ to ‘high’ intensity interventions occurred in more recent years. However, ‘participation’ has been consistently low in a majority of the studies over the last 15 years. One of the reasons for this may be the fact that program planners focus on the content of the interventions instead of balancing the content with the experience of the participant—that is, on the ‘participation’ dimension and the engagement factor that connects the participant with the intervention.

Future translational research in this field needs to invest in designing recruitment more carefully to ensure high program reach; examining factors that optimize engagement and retention in the structured lifestyle programs; and maximizing adherence to the long-term behaviour changes [59].

### Limitations

Several studies did not provide relevant numeric data to allow the calculation of PIPE coefficients. Hence we were unable to compare the overall program impact of the included studies. Additionally, any successfully translated diabetes program should ideally be accessible to those most in need and should have some clear relationship with the health care delivery system, and we were not able to evaluate these two elements in most of the studies included in this review.

In our analysis of implementation, we only considered standard sessions and have not included the extracurricular or optional activities offered by some of the programs that may have impact on the future adoption of these kinds of programs. This was mainly due to the heterogeneity in how the extracurricular activities were organized and also how they were reported. However, we do acknowledge that these ‘spinoff’ activities are likely to be very important for sustainability and the wider diffusion of programs, their maintenance, and sustainability.

Although there has been a lot of emphasis on the need for translating T2DM programs in low and middle income countries where the majority of people in the world at high risk of diabetes and its progression live [60], we did not find any studies from such countries that met our eligibility criteria. The Kerala Diabetes Prevention Program [61] in India is one of the first such implementation trials to evaluate a peer-led, group-based lifestyle intervention program (based on the Finnish GOAL study) [41] among individuals at high risk of developing T2DM in rural India. The trial is currently being implemented and will provide important reference to the translation of diabetes prevention programs in India and similar countries.

Also, we did not find any studies that have previously utilized the PIPE Impact Metric framework in diabetes prevention. More research is needed to understand and apply the few but essential elements of the PIPE model to measure the overall public health impact of diabetes prevention interventions.

### Implications for practice

The original US-DPP [2] and Finnish-DPS [3] efficacy trials demonstrated that lifestyle intervention is an effective way to reduce the risk of T2DM in high-risk adults. However, achieving better translation of these programs still remains challenging after 15 years of research. Our findings suggest that program planners and implementers should aim to design high-intensity program with frequent contacts if the primary target is weight loss. However, if the primary aim is diabetes risk reduction, this can also be achieved with lower frequency of contacts but with a program duration of at least 12 months. With this program design, program planners should expect only low or moderate weight loss. To have a broader public health impact, programs with lower frequency of contacts but with a program duration of at least 12 months might be more feasible but this requires program strategies that simultaneously address both penetration and participation. Future translational research needs to identify effective recruitment and program implementation strategies for targeting both reach and program participation that also emphasizes long-term program adherence.

To improve the translation of diabetes prevention programs in real-world settings, we suggest a more rigorous reporting of program elements and components to evaluate these programs to assess the practical value [27] of the diabetes prevention programs. In particular, more detailed reporting on the four key PIPE Metric components will provide important insights and has the potential to facilitate increased uptake of T2DM prevention programs worldwide. We also suggest a greater consistency of reporting main outcomes and a standardization of reporting criteria for translational diabetes prevention programs implemented in real-world settings.

## Conclusions

Our findings based on program implementation over the period of 15 years suggest that while a high-frequency intervention plays an important role in achieving high weight loss outcomes, programs with ‘low’-intense interventions have also shown high reductions in the incidence of T2DM. This suggests that even when the effectiveness of an intervention is moderate in terms of weight loss, it can have a profound impact on the development of a disease at the population level—provided enough effort is put into guaranteeing high penetration and participation as well. From a translation perspective, not many studies provide the necessary information to estimate the overall impact of such programs. Key elements of the PIPE Impact Metric are not routinely reported in many published implementation trials of diabetes prevention which therefore reduces their utility for information resource allocation and ‘real-world’ implementation. More rigorous evaluation methods are required to better understand the factors that influence the likely success of such interventions in the future.

## Additional files

Additional file 1: **Search Strategy.** Search strategy used for identifying included studies. (PDF 371 KB) Additional file 2: Table S4–Table S7 **Detailed scoring of all included studies based on the elements of the PIPE Impact Metric framework.** (PDF 772 KB)
