# Supplementary material for: A systematic review of real-world diabetes prevention programs: learnings from the last 15 years
Source: Implement Sci. 2015 Dec 15;10:172. doi: 10.1186/s13012-015-0354-6 (PMC4681022; doi:10.1186/s13012-015-0354-6)
Supplement: Additional file 1: — Search Strategy. Search strategy used for identifying included studies. (PDF 371 KB) [file 13012_2015_354_MOESM1_ESM.pdf]

A systematic review of real-world diabetes prevention programs: learnings from the last 15 years.

Search Strategy

PubMed (10/Feb/2014)

1.	Diabetes[Title/Abstract]	320,653
2.	(prevention[Title/Abstract]) OR preventing[Title/Abstract]	452,289
3.	(program[Title/Abstract]) OR programme[Title/Abstract]	350,373
4.	(intervention[Title/Abstract]) OR interventions[Title/Abstract]	512,588
5.	#3 OR #4	815,917
6.	(implementation[Title/Abstract]) OR implementing[Title/Abstract]	143,739
7.	(translation[Title/Abstract]) OR translating[Title/Abstract]	89,807
8.	#6 OR #7	231,498
9.	#1 AND #2 AND #5 AND #8	507

Web of Science (10/Feb/2014)

TOPIC: (diabetes prevention) ANDTOPIC: (intervention OR program)
AND TOPIC: (implementation OR translation) NOT TOPIC: (type 1) NOT TOPIC: (kidney)
Timespan=2001-2014. Indexes=SCI-EXPANDED, SSCI, CCR-EXPANDED, IC.

- All (365)

MEDLINE (10/Feb/2014)

1. Diabetes.mp. or Diabetes, Gestational/ or Diabetes Complications/ or Diabetes Mellitus, Experimental/ or exp Diabetes Mellitus/ or exp Diabetes Mellitus, Type 2/ 399,338

2.	exp Primary Prevention/ or Prevention.mp. or exp Secondary Prevention/	442,300
3.	Diabetes Mellitus/ or Preventing	196,772
4.	#2 OR #3	617,050
5.	program or programme or intervention or interventions	776,170
6.	implementation or implementing or translation or translating	203,957
7.	#1 AND #4 AND #5 AND #6	846

CENTRAL (17/Feb/2014)

1.	Diabetes	28,504
2.	Prevention OR preventing	123,018
3.	Program or programme	54,085
4.	Intervention or interventions	104,485
5.	#3 OR #4	132,644
6.	Implementation or implementing	13,155
7.	Translation or translating	6,209
8.	#6 OR #7	18,076
9.	#1 AND #2 AND #5 AND #8	1,366
10.	Trials	180

EMBASE (10/Feb/2014)

1.	'diabetes'/exp	480,005
2.	'prevention'/exp	2,311,308
3.	'program'	879,533

4.	‘intervention’	495,471
5.	#3 OR #4	1,314,953
6.	Implementation	143,654
7.	Translation	666,965
8.	#6 OR #7	808,155
9.	#1 AND #2	82,216
10.	#9 AND #5 AND #8	1,094

Total Records Found:

1. PubMed (507)
2. Web of Science (365)
3. MEDLINE (846)
4. CENTRAL (180)
5. EMBASE (1,094)

Total = 2,992
