# Supplementary material for: A systematic review of real-world diabetes prevention programs: learnings from the last 15 years
Source: Implement Sci. 2015 Dec 15;10:172. doi: 10.1186/s13012-015-0354-6 (PMC4681022; doi:10.1186/s13012-015-0354-6)
Supplement: Additional file 2: Table S4–Table S7 — Detailed scoring of all included studies based on the elements of the PIPE Impact Metric framework. (PDF 772 KB) [file 13012_2015_354_MOESM2_ESM.pdf]

# A systematic review of real-world diabetes prevention programs: learnings from the last 15 years.

## Table S4 – Table S7 [Detailed scoring of all included studies based on the elements of the PIPE Impact Metric framework]

## Supplementary Table 4 – Scoring of all included studies (PENETRATION)

| Penetration | | | | |
|---|---|---|---|---|
| **Author Year** | **Study ID** | **Scoring** | **Target Population** | **Description** |
| Mensink et. al. 2003 [35] | SLIM | High | 6108 | Study population Adults at risk of diabetes.<br>ALL eligible subjects with high risk for glucose intolerance (n = 6108) from a cohort representing general population were contacted. 6108 / 6108 = 1 (100%) |
| Kosaka et. al. 2005 [62] | Japanese DPP | NAC | Not Reported | Since 1983 the Health Medical Centre in Toranomon Hospital has been conducting a health-screening program, mostly for government employees. We randomly selected subjects (at risk) from among examinees in 1990-92. |
| Oldroyd et. al. 2006 [63] | Newcastle Lifestyle Intervention | NAC | Not Reported | Individuals with IGT identified from the three sources of recruitment (n = 498). Potential participants were sent a letter inviting them to take part with a pre-paid reply slip. |
| Absetz et. al. 2007 [41] | GOAL LIT | NAC | Not Reported | Participants were patients (age 50–65 years) with already-identified risk factors (obesity, hypertension, elevated blood glucose, or lipids) presenting at local primary health care centres and identified by physicians and nurses. Number of patients approached not reported. |
| Bo et. al. 2007 [36] | Italian Trial | High | 1877 | ALL subjects aged 45-64 from 6 family physicians, representative of the local Health Districts, were contacted (n = 1877) → 1877 / 1877 = 1 (100%) |
| Davis-Smith et. al. 2007 [39] | DPP (church-based) | Moderate | 407 | Study population is Adults at risk of diabetes. An African-American Church in a rural Georgia town with a population of 8,040 was identified. The church's roster included 407 members. The church had Sunday attendance of approximately 150 adults. ALL adults were invited to complete a diabetes risk assessment.<br>150 / 407 = 0.368 (36.8%) |
| Laatikainen et. al. 2007 [64] | Greater Green Triangle (GGT) | NAC | Not Reported | Study population 'Adults at risk of diabetes'.<br>Participants were patients presenting at local General Practices and were screened opportunistically by study nurses in reception and waiting areas. Over 1500 people approached. |
| Ackermann et. al. 2008 [65] | DEPLOY | NAC | Not Reported | Study population is Adults at risk of diabetes. Mail invitations to 7,500 randomly selected households. Actual target population NR. |
| Boltri et. al. 2008 [66] | DPP (church-based | NAC | Not Reported | Study population is Adults with prediabetes. Risk assessment was administered on ALL church attendees aged $\geq$18 years on two successive Sundays. |

| **Penetration** | | | | |
|---|---|---|---|---|
| **Author Year** | **Study ID** | **Scoring** | **Target Population** | **Description** |
| Payne et. al. 2008 [67] | BDPPI | NAC | Not Reported | Total Population of city of Ballarat = 86,977. Target pop for this study not reported. |
| Kramer et. al. 2009 [68] | GLB (2007 – 2009) | NAC | 15000 | Study population is Adults with prediabetes. The two research practices were located in suburban areas of Pittsburgh; one practice had a patient base of approx. 5000 and the other approx. 10,000. Potential participants learned about the GLB program through newspaper announcements and posted flyers, or were referred by their physician. Physician referral with permission for physical activity was required for all participants. A total of 74 referrals were received. |
| Kulzer et. al. 2009 [69] | PREDIAS | NAC | Not Reported | Not Reported |
| Penn et. al. 2009 [70] | EDIPS-Newcastle | NAC | Not Reported | Recruitment was by referral from primary care physicians who identified eligible people likely to be at risk, from their primary care databases and invited them to participate. |
| Almeida et. al. 2010 [71] | Colorado Weight Loss Intervention | NAC | Not Reported | Potential participants were individuals with newly diagnosed prediabetes. Existing electronic medical records were accessed to create a cohort of 14,379 people with IFG. Eligible participants 12,468. The paper does not explicitly tell whether and how all these were contacted, however it does give the impression that they were. It says "Of the 12,468 members eligible for participation in the small-group sessions, 1,030 took part" – but did all eligible know of the study? |
| Makrilakis et. al. 2010 [72] | DE-PLAN Greece | NAC | Not Reported | Study population is" Adults at risk of diabetes. "The staff of six primary care centres were asked to distribute the FINDRISC questionnaire to ALL people without diabetes visiting their centres within a time period.<br><br>In the occupational setting, a 'day for diabetes prevention' was arranged at each of the six companies. On that day, doctors of the investigators' team visited the company and distributed the FINDRISC questionnaires to all employees working on that specific date. The response rate was substantially higher (approximately 80%). In this case the questionnaires were completed on site and the employees were informed about the DE-PLAN project by the investigators themselves. |
| Parikh et. al. 2010 [73] | Project HEED | NAC | Not Reported | The board developed several recruitment strategies that members of the board and study personnel implemented at community sites and events, such as churches, social service agencies, senior centres, and health fairs. |
| Vanderwood et. al. 2010 [45] | Montana CDDP | NAC | Not Reported | For this pilot study, the goal for each of the 4 sites was to recruit and enrol 100 eligible participants into the program. Each site used various strategies to recruit potential participants, incl. contacting local physicians, advertising through media, recruiting through local employers, work sites, churches etc. As a result, 355 participants were enrolled in the program. |

| Penetration | | | | |
|---|---|---|---|---|
| **Author Year** | **Study ID** | **Scoring** | **Target Population** | **Description** |
| Vermunt et. al. 2010 [74] | APHRODITE | NAC | Not Reported | All 48 GPs from an association of primary care practices agreed to participate. ~99% of the inhabitants are registered with a GP. Each GP selected a random sample of his/her patients aged >40 and <70 years (n = 16,032). |
| Boltri et. al. 2011 [75] | DPP (church-based) | NAC | Not Reported | Study population is Adults with prediabetes. ALL participants in 5 churches on two subsequent Sundays were invited. 5 churches with membership in each 200-400, i.e., target population 1000-2000. All church attenders 442, eligible for study 400. 442/1000 = 44%; 442/2000 = 22%<br><br>High-risk adult members of the church were identified using the 7-question DRA tool. Two weeks prior to implementing the DRA, weekly announcements were placed in the Sunday bulletin detailing the dates of the DRA. The DRA was performed during 2 successive Sunday services...The DRA was distributed to every adult congregant, collected on the same day… |
| Gilis-Januszewska et. al. 2011 [76] | DE-PLAN Poland | NAC | Not Reported | Study population is ‘Adults at risk of diabetes. ‘Advertisements were placed alongside 800 self-screening questionnaires in the GP's waiting rooms. In addition, those patients with known risk factors were approached by nursing and medical staff. |
| Katula et. al. 2011 [77] | HELP PD | NAC | Not Reported | Study population Adults with prediabetes.<br>"Standard recruitment strategies were employed, including mass mailing at targeted addresses, direct provider referral, and community events." Between 500-2000 mailings per week (over 24-mo recruitment period) but total N not reported. |
| Kumanyika et. al. 2011 [48] | Think Health! | NAC | Not Reported | Study participants identified from billing lists and clinician referrals. Local university- or hospital-affiliated primary care practices, as well as independent individual or group physician practices. All clinical sites were asked to commit to recruiting at least 50 participants. Number of sites not reported. |
| Nilsen et. al. 2011 [42] | Nilsen et. al. | NAC | Not Reported | All general practitioners in the four nearest municipalities to the hospital were each supplied with ten FINDRISC-questionnaires by post, asked to use them on patients at risk for T2D and were requested to refer individuals aged 18-64 with a FINDRISC-score > 9 to the hospital. |
| Penn et. al. 2011 [43] | NLNY | NAC | Not Reported | The town of Middlesbrough was chosen for the pilot because of the large proportion of the target population and high prevalence of T2D. Target population not reported. Social marketing campaign attracted 271 individuals. |
| Ruggiero et. al. 2011 [78] | HLP | NAC | Not Reported | Study population Adults at risk of diabetes.<br>The participants were recruited during free health screenings (n = 1162 people were assessed at 20 study-sponsored screenings) held in the community at various locations incl. public schools, community/ family service centres, a hospital, and health centres. Posters and flyers were delivered to schools, community centres and local businesses. |

| Penetration | | | | |
|---|---|---|---|---|
| **Author Year** | **Study ID** | **Scoring** | **Target Population** | **Description** |
| Sakane et. al. 2011 [79] | Japanese Study | NAC | 10000+ | Study population Individuals with impaired glucose tolerance. Using the data from health check-ups, those who met the eligibility criteria were extracted. It was roughly estimated that there were more than 10,000 people with borderline hyperglycemia at the 32 collaborative centres. Each centre recruited study candidates using posters, through flyers, and by word of mouth during the time period of the study. |
| Costa et. al. 2012 [40] | DE-PLAN-CAT | Low | 315703 | Study population is 'Adults at risk of diabetes. Participating centres (n=18) covered all primary care services for 315,703 inhabitants (4.5% of the population in Catalonia).<br>Participants invited for screening = 2,547<br>2547 / 315703 = 0.0080 **(0.8%)** |
| Janus et. al. 2012 [46] | pMDPS | NAC | Not Reported | The original MDPS was designed to recruit 1,300 individuals. For this version of MPDS (pMDPS), 99 individuals were recruited during 2009-10. Target pop. not reported. |
| Kanaya et. al. 2012 [50] | LWBW | NAC | Not Reported | We focussed on community-dwelling adults in 4 districts low-income neighbourhoods in northern California cities. Recruitment began with community-based, educational outreach to identify individuals at risk of diabetes. |
| Lakerveld et. al. 2012 [37] | Hoorn Prevention Study | High | 12,000 | Study population Adults at risk of diabetes and/or CVD.<br>A total of 8193 men and women aged 30-50 years living in several municipalities were invited to participate in a selective screening procedure by mail. The target group was approached after identification of date of birth and absence of diabetes and known CVD from 12 participating general practices.<br><br>The target population in the protocol paper was 12,000. 8,193 men and women aged 30 to 50 years living in several municipalities in a semirural region (West-Friesland) of the Netherlands were invited to participate in a selective screening procedure by mail.<br>8193/12,000 = 68% |
| Ockene et. al. 2012 [80] | LLDPP | NAC | Not Reported | The primary recruitment outreach method entailed drawing from the GLFHC patient panel by identifying potentially eligible patients who received a mail letter of invitation… Additional outreach methods included announcements on local radio and television stations, newspaper advertisements, and mailings to non-GLFHC physicians. Patients were also screened as walk-ins if they learned of a screening event via word-of-mouth or community outreach. |
| Piatt et. al. 2012 [81] | GLB (2005-2008) | NAC | Not Reported | Study population 'Adults with metabolic syndrome'.<br>Adults age $\geq$18 from the 11 target neighborhood were eligible to be screened for metabolic syndrome. Recruitment involved posting flyers, info. in the local newspaper and on the local cable broadcast channel. |
| Jiang et. al. 2013 [82] | SDPI-DP | NAC | Not Reported | SDPI-DP identified potential participants (n = 18,134) mainly through community events such as health fairs but also recruited participants from local clinics or by provider referral. |

| **Penetration** | | | | |
|---|---|---|---|---|
| **Author Year** | **Study ID** | **Scoring** | **Target Population** | **Description** |
| Ma J et. al. 2013 [38] | E-LITE | High | 3439 | Study population overweight / Obese adults with increased cardio-metabolic risk.<br>“We identified overweight or obese, at-risk adults receiving care from the 21 active primary care providers at the study clinic site using the electronic health record system.<br>Potential participants identified in the EHRs and approved by primary care providers = 3439<br>Contact attempted for screening = 2391<br>2391 / 3439 = 0.695 (69.5%) |
| Duijzer et. al. 2014 [49] | SLIMMER | NAC | Not Reported | Participants for the pilot study were recruited from August to September 2010 by 3 general practitioners from their patient registration database. Each GP selected a random sample of patients from the database aged 40 through 65 years with IFG. |
| Sepah et. al. 2014 [47] | Prevent | NAC | Not Reported | Participants were recruited from online advertisements, seeking individuals with a self-reported clinical diagnosis of pre-diabetes occurring within the past year. |
| Zyriax et. al. 2014 [34] | DELIGHT | High | 3000 | ALL (n = 3000) employees from five companies were approached through flyers, intranet, or works meeting to invite for further check-up if their waist circumference was above certain limit.<br>3000/3000 = 1 (100%) |
| Savas et. al. 2015 [44] | IGT Care Call | NAC | Not Reported | Seven GP practices with IGT registers participated in the project. They referred people with IGT diagnosed by a 2-hour oral glucose tolerance test. |

*Note: NAC = Not Able to Calculate*

## Supplementary Table 5 – Scoring of all included studies (IMPLEMENTATION)

| Implementation | | | | | |
|---|---|---|---|---|---|
| **Author Year** | **Study ID** | **Frequency** | **Duration** | **Fidelity** | **Description** |
| Mensink et. al. 2003 [35] | SLIM | Low | High | Low | **Frequency:** Three individual and one group session given by dietitian during 1 year + participants were encouraged to participate in the exercise program (consisting of once-a-week free exercise sessions). 4-6 / 22 = 18.2%-27.3%<br>**Duration:** High ($\geq$ 12-months)<br>**Fidelity:** "The intervention program consists of a dietary and physical activity part, with visits scheduled at regular intervals throughout the study." |
| Kosaka et. al. 2005 [62] | Japanese DPP | Low | High | Low | **Frequency:** To achieve the body weight objective, a set of questions and instructions were repeated every 3–4 months at each hospital visit. These means of intervention were repeatedly explained at hospital visits every 2–3 months.<br>4-5/22 = 18.2%-22.7%<br>**Duration:** High Up to 4 years = $\geq$ 12-months<br>**Fidelity:** Since they were following the above mentioned set of instructions in every hospital visit, should that be considered as a measure of Fidelity (standard curriculum)? |
| Oldroyd et. al. 2006 [63] | Newcastle Lifestyle Interventio n | Moderate | High | NAC | **Frequency:** 2 baseline motivational interviews; 6 individual 15-20 minutes review appointments in 6 months; and 1 review appointment at 9 months. 9/22 = 40.9%<br>5 review appointments at two monthly intervals between 12 & 24 months.<br>**Duration:** $\geq$ 12-months<br>**Fidelity:** Not reported |
| Absetz et. al. 2007 [41] | GOAL LIT | Moderate | Moderate | Moderate | **Frequency:** Six 2-hourly group counselling sessions over 8 months. 6/22=27.3%<br>**Duration:** 8 months<br>**Fidelity:** Program components were more frequently added (40%) than omitted (28%). |
| Bo et. al. 2007 [36] | Italian Trial | Low | High | High | **Frequency:** 5 sessions of at least 60 minutes covering diet, exercise, and behavior modifications were held, the first was a one-to-one meeting and was followed by group sessions based on behavioral counseling and focusing on practical lifestyle tips. 5/22=22.7%<br>**Duration:** 5 sessions of at least 60 minutes covering diet, exercise, and behavior modifications were held. $\geq$ 12-months<br>**Fidelity:** To minimize the potential lack of fidelity, the professional health care providers (n=8) were assigned to the sessions in a quasirandomized mode (i.e., a scheduled rotation of experts among sessions to assure a balanced intervention for all subjects), and the delivered information was controlled. Each provider checked messages previously given and patients' expectations. The sessions had a flexible structure, sensitive to cultural differences and patient expectations, that combined a structured core (common topics, recommendations, and educational leaflets) with flexibility in verbal presentation tailored to requirements and retention capacity. |

| Implementation | | | | | |
|---|---|---|---|---|---|
| **Author Year** | **Study ID** | **Frequency** | **Duration** | **Fidelity** | **Description** |
| Davis-Smith et. al. 2007 [39] | DPP (church-based) | Low | Low | Moderate | **Frequency:** A six-session program was designed from the 16-session intensive lifestyle arm of the DPP. The six sessions were presented over a seven-week period following a schedule determined by the participants. Advance letters were mailed to remind the participants of each upcoming session and to encourage them to meet the goals of the previous session. Participants were supported with calls, letters and reminders of sessions. Number of advance letters and calls not specified. 6/22 = 27.3%<br>**Duration:** Seven-week period<br>**Fidelity:** The research team selected six of the NIH-DPP's 16 sessions for this pilot study based on information from prior focus groups with church members. The 16-session DPP has three themes: nutrition, physical activity and behaviour change. Each theme is the main focus of four sessions. We identified two sessions from each of these three categories to make up the six sessions. The material was modified to be used in a church-based group setting from its original design for individual use with a lifestyle coach. A nurse educator and physician performed the modification, adapting the information to a group setting. Each session was led by volunteer healthcare professionals. The session leaders attended a 60-minute training session led by the research team on how to present the lifestyle balance curriculum to the church community. |
| Laatikainen et. al. 2007 [64] | Greater Green Triangle (GGT) | Low | Moderate | Low | **Frequency:** Six structured 90-minutes group sessions delivered during an 8 months period using the Health Action Process Approach. 6/22=27.3%<br>**Duration:** 8 months<br>**Fidelity:** The intervention model used in the study was based on the diabetes prevention project in the Finnish GOAL study. |
| Ackermann et. al. 2008 [65] | DEPLOY | High | High | High | **Frequency:** The intervention core curriculum involved 16 classroom-style meetings. Program sessions lasted 60–90 minutes, and the entire core curriculum was delivered over 16–20 weeks. …maintenance activities following the core curriculum sessions involved monthly, large-group meetings.<br>22/22=100%<br>**Duration:** $\geq$12-months<br>**Fidelity:** The study was based on the US-DPP model. Several approaches were used to ensure that intervention sessions were delivered with fidelity to the DPP model. These approaches were based on similar strategies used during the DPP, and were developed by a training core of DPP project staff, YMCA personnel, and the principal investigator. Quality assurance began with the structured training and certification process described above, adapted from the DPP manual of operations. |

| Implementation | | | | | |
|---|---|---|---|---|---|
| **Author Year** | **Study ID** | **Frequency** | **Duration** | **Fidelity** | **Description** |
| Boltri et. al. 2008 [66] | DPP (church-based | High | Low | Moderate | **Frequency:** Sixteen weekly group sessions conducted over 4 months. 16/22=72.7%<br>**Duration:** 4 months<br>**Fidelity:** Volunteer medical personnel with diabetes prevention experience attended a training session that included education on the goals of the DPP, how to use the DPP materials, and information on leading the 60- to 90-minute long group sessions.<br>At the beginning of each session workbook materials were distributed. Each session started with a prayer usually led by a deacon or minister. The group leader then led a group discussion for 60 to 90 minutes that was designed to disseminate the content of the DPP and encourage peer support. Discussion questions concerned<br>DPP-related topics relevant to the session. |
| Payne et. al. 2008 [67] | BDPPI | High | High | Moderate | **Frequency:** The intervention included a 6-week group self-management education program, a gymnasium-based or home-based 12-week resistance training program, and a 34-week maintenance program.<br>During week 1 – 6, the intervention included six 1.5 hours group self-management education program.<br>During weeks 7–18, participants were assigned to either a gym-based or a home-based 12-week resistance training program.<br>The intervention included a 34-week maintenance program. Participants were encouraged to continue the recommended regimen and to attend three 2-hour group reinforcement sessions. (3 * 2 = 6 hours) and (6 / 1.5 = 4 sessions)<br>They were also sent two newsletters containing self-management, healthy eating, and physical activity advice.<br>6+12+4+0.5 = 22.5/22 = 102%<br>**Duration:** $\geq$ 12-months<br>**Fidelity:** The BDPPI methodology was based on National Evidence Based Guidelines for the Management of Type 2 Diabetes Mellitus developed by the Australian National Health and Medical Research Council (NHMRC). |
| Kramer et. al. 2009 [68] | GLB (2007 – 2009) | High | Moderate | Moderate | **Frequency:** GLB program participants attended 12 weekly 1-hour sessions delivered over 12-15 weeks. Participants in Phase 2 were offered the opportunity to attend monthly support meetings for 9 months after completion of the intervention.<br>12 + 9 = 21 → 21/22= 95.4%<br>**Duration:** 9 months (Phase 2)<br>**Fidelity:** The DPP lifestyle intervention was adapted to a 12-session group-based GLB program. A model for training and support mirroring that of the DPP was developed for prevention professionals administering the program. |

| Implementation | | | | | |
|---|---|---|---|---|---|
| **Author Year** | **Study ID** | **Frequency** | **Duration** | **Fidelity** | **Description** |
| Kulzer et. al. 2009 [69] | PREDIAS | Moderate | Low | Moderate | **Frequency:** The prevention program consisted of 12 lessons lasting 90 min each. During the first 8 weeks, eight core lessons were given with one per week; the last four lessons were bimonthly booster lessons. 12/22=54.5%<br>**Duration:** 4 months<br>**Fidelity:** The PREDIAS program, which is based on self-management theory, was conducted in small groups (median size seven people). PREDIAS was delivered by either diabetes educators or psychologists. The program comprised a set of transparencies for the lessons and a curriculum for the prevention manager. |
| Penn et. al. 2009 [70] | EDIPS-Newcastle | Moderate | High | NAC | **Frequency:** A 30-min session immediately following randomisation and two weeks later, then monthly for the first three months and every three months thereafter up to five years. 2+3+3 = 8/22 = 36.4%<br>**Duration:** 5 years<br>**Fidelity:** Not clear |
| Almeida et. al. 2010 [71] | Colorado Weight Loss Interventio n | Low | Low | NAC | **Frequency:** A single 90-minute small-group session.<br>**Duration:** 1 session only.<br>**Fidelity:** Not reported |
| Makrilakis et. al. 2010 [72] | DE-PLAN Greece | Low | Moderate | Low | **Frequency:** The 1-year intervention programme consisted of six sessions (1 hour each) held by a registered dietitian at the area of the participants' residence or work. Groups of 6–10 persons were constructed.<br>6/22=27.3%<br>**Duration:** 12 months<br>**Fidelity:** According to the general DE-PLAN protocol, each centre of the participating countries was allowed to follow any intervention strategy—assisted self-managed, group-based or individual-based consultation—with the objective of achieving better understanding of the disease risk from the participants and of building up motivation for an intention to change lifestyle. In our study, group-based consultation interventions were chosen, as they were deemed to be more conveniently implemented, more cost-effective and efficacious from the participants' standpoint. |
| Parikh et. al. 2010 [73] | Project HEED | Moderate | Low | Moderate | **Frequency:** A workshop consisting of eight 1.5-hour sessions over 10 weeks. 8/22=36.4%<br>**Duration:** 10 weeks<br>**Fidelity:** Project HEED's curriculum followed self-efficacy theory; contained simple, actionable messages; was easily taught by lay leaders; and focused on enhancing self-efficacy to make lifestyle changes. We reviewed the curriculum with scientific and peer education experts, tested it with English (n=6) and Spanish (n=12) speakers, and revised accordingly. |

| Implementation | | | | | |
|---|---|---|---|---|---|
| **Author Year** | **Study ID** | **Frequency** | **Duration** | **Fidelity** | **Description** |
| Vanderwood et. al. 2010 [45] | Montana CVD & DPP | High | Moderate | Moderate | **Frequency:** Sixteen weekly group sessions, and six monthly group sessions. 22/22=100%<br>**Duration:** 10 months (16 weekly + 6 monthly)<br>**Fidelity:** DPP Based. The DPHHS funded eight health care facilities with recognized diabetes self-management education programs beginning in 2008. Sites used trained health professionals as lifestyle coaches to provide the 16-session core followed by 6 monthly after core sessions. |
| Vermunt et. al. 2010 [74] | APHRODITE | Moderate | High | NAC | **Frequency:** Eleven consultations of 20-min over 2.5 years, five 1-h group meetings and, 1-h personal consultation with the dietician.<br>8 of these sessions within 12 months 8/22=36.4%<br>**Duration:** $\geq$ 12-months<br>**Fidelity:** Not clear |
| Boltri et. al. 2011 [75] | DPP (church-based) | Low (2 churches) High (3 churches) | Low | Moderate | **Frequency:** This pilot study was carried out in a total of 5 churches. Two group-based programs, a 6-session program and a 16-session program, were developed.<br>Two churches participated in the 6-session diabetes prevention program and 3 churches participated in the 16-session program.<br>6/22=27.3%<br>16/22=72.7%<br>**Duration:** $\leq$ 6-months<br>**Fidelity:** The intervention used in this study was modeled after the lifestyle intervention arm of the NIH-DPP… The team modified the lifestyle balance curriculum of the DPP from an individual based design to a group-based design that is culturally appropriate for implementation in an African American church setting… The new format was test piloted in a small group setting using a research team that included African American members of local churches. Minor modifications were made based on feedback from church participants who completed the program. |
| Gilis-Januszewska et. al. 2011 [76] | DE-PLAN Poland | Moderate | Moderate | Low | **Frequency:** The intensive intervention part lasting for four months consisted of 10 group sessions (10–14 people each) on lifestyle changes, diet and physical activity education and was followed by a 6-month continuous part including six telephone motivation sessions and two motivation letters sent to the study participant.<br>10 + (6*0.5 = 3) + (2*0.25 = 0.5) = 13.5 → 13.5/22=61.4%<br>**Duration:** 10 months<br>**Fidelity:** The intervention curriculum was created with written materials concerning the basic information about diabetes, diabetes prevention, diet, a diet examples booklet and information about physical activity (available at www.image-project.eu) |

| Implementation | | | | | |
|---|---|---|---|---|---|
| **Author Year** | **Study ID** | **Frequency** | **Duration** | **Fidelity** | **Description** |
| Katula et. al. 2011 [77] | HELP PD | High | High | High | **Frequency:** Participants met weekly for CHW-led group sessions during the first 6 months. Participants also received three personalized consultations with a registered dietician (during months 1, 3, and 6). During months 7–12, participants received two scheduled contacts with the CHW each month, one group session and one telephone contact. 26 weekly sessions + 3 personalized consultations + 3.5 schedule contacts each months for 6 months (3.5 * 6 = 21) = 50 sessions → 50/22=227%<br>**Duration:** $\geq$ 12 months<br>**Fidelity:** The HELP PD project was designed to translate the methods of the DPP into the community via key modifications to enhance feasibility and dissemination..<br>Intervention content standardization was supported by a DVD series developed by the research team. |
| Kumanyika et. al. 2011 [48] | Think Health! | Moderate | Moderate | Moderate | **Frequency:** For Think Health! the initial intervention period was extended to 1 year rather than the 24 week core in DPP. The moderate-intensity Think Health! condition ("Basic Plus") offered about 2–4 hour total contact over an entire year (10–15 min sessions every 4 months with the Primary Care Providers (PCP) and similarly brief contacts with a Lifestyle Coaches (LC) monthly.<br>3 session with PCP + 8? Contacts with LC (11 / 22 = 50%)<br>**Duration:** 12 months<br>**Fidelity:** The 16 core DPP sessions were modified based on a prior DPP adaptation.<br>Some DPP core content was put in supplemental handouts, and some was shifted to the maintenance period in the second year of treatment. The amount of content conveyed per session was reduced to a set of key points that could be summarized in a two-page handout. These handouts were packaged in participant manuals, which also included a CD with audio narration of the first 12 sessions…<br>The year 1 treatment protocol was implemented as intended, although not all treatment visits were completed with all patients due to nonattendance.<br>...we do not have data on time spent in counseling or quality of PCP or LC counselling provided. We also cannot assess possible differences in how PCPs counseled participants in Basic vs. Basic Plus (based on their inadvertent knowledge of the participant's randomization assignment) and do not know the degree of individual tailoring that occurred within counseling sessions. Although materials were standardized, PCPs were encouraged to use their own styles of working with patients generally and with the particular patient. |

| Implementation | | | | | |
|---|---|---|---|---|---|
| **Author Year** | **Study ID** | **Frequency** | **Duration** | **Fidelity** | **Description** |
| Nilsen et. al. 2011 [42] | Nilsen | High | High | NAC | **Frequency:** Participants consulted the study physician at six, twelve and eighteen months after randomisation. The study physician used elements of motivational interviewing during these consultations. In addition, the group participated in a group based program, one day (five hours per day) each week for six weeks and a new gathering after twelve weeks. An individual 30-minutes consultation with a nurse or ergonomist completed the intervention one month after the last group meeting.<br>2 contact for motivational interviewing at 6 and 12-mo<br>Each 5 hour session = 2 sessions i.e. 2 * 6 = 12 sessions<br>1 individual consultation<br>(2 + 12 + 1 = 15) 15/22 = 68%<br>**Duration:** >12-mo<br>**Fidelity:** Not clear |
| Penn et. al. 2011 [43] | NLNY | High | Moderate | Low | **Frequency:** NLNY trainers delivered a 10-week programme of twice weekly 1.5 h sessions to groups of 15–20 participants. Each NLNY session comprised a supervised PA or, on two or three occasions within each 10-week programme, a cookery session, followed by a reflective discussion that covered PA, nutrition, weight management and strategies for behaviour change. Monthly NLNY newsletters with information, advice and recipes were available to participants, mostly online.<br>At the end of the 10-week programme, participants who had completed ≥80% attendance received an 'access to leisure' card that enabled free leisure service use for 12 months. After the programme, ongoing support with regular mobile phone text message and email reminders, 'drop-in' activity sessions and encouragement to join in local events such as organised walks and runs continued up to the assessment at 12 months of follow-up.<br>10 * 2 = 20 sessions + mobile phone, text messages and email reminders; drop-in activity sessions etc.<br>20+/22 = >100%<br>**Duration:** 12 months<br>**Fidelity:** Nutritional information…was in line with the DPS protocol and NICE guidance. |
| Ruggiero et. al. 2011 [78] | HLP | High | Moderate | Moderate | **Frequency:** The MTC HLP was modeled after the DPP's successful 1-year intensive lifestyle program and included a core program with weekly sessions that shifted to monthly sessions for the "after core" program.<br>22/22=100%<br>**Duration:** 12 months<br>**Fidelity:** The MTC HLP curriculum closely followed the DPP's intensive lifestyle curriculum (Spanish version). |

| Implementation | | | | | |
|---|---|---|---|---|---|
| **Author Year** | **Study ID** | **Frequency** | **Duration** | **Fidelity** | **Description** |
| Sakane et. al. 2011 [79] | Japanese Study | Moderate | High | Moderate | **Frequency:** During the initial six months, 4 group sessions were conducted with each session lasting 2 or 3 hours. The individual session was conducted biannually during the three years with each session lasting 20 to 40 minutes. To reinforce the intervention, between-visit contact by fax was also made monthly during the initial twelve months.<br>4 group sessions; 2 individual sessions; and 12 monthly contacts through fax = (6 * 0.25 = 1.5 sessions)<br>4 + 2 + 1.5 = 7.5 / 22 = 34%<br>**Duration:** > 12 months<br>**Fidelity:** Compared with the DPS and DPP, the present study had a less intensive intervention. The majority of the public health nurses, reflecting the real world primary healthcare setting, did not have special training in lifestyle modifications. At a feasible level, they carried out the intervention using the protocol and educational materials provided by the study group. As a rule, the same study nurse carried out the interventions on the same participant during the study. But this was not always possible due to a personnel change at the collaborative center. |
| Costa et. al. 2012 [40] | DE-PLAN-CAT / PREDICE | Low | High | NAC | **Frequency:** The intervention consisted of two steps (initial and further reinforcement). The intensive intervention consisted of a six-hour educational program (divided in 2 to 4 sessions) to be performed either individually or in small groups (5 to 15 people). Participants were reminded (telephone call) about the date and hour of their next group session to ensure compliance, and every 6-week (minimum) they were contacted (also by telephone). In 2 out of 8 centres SMS sending was preferred for continuous intervention.<br>4 sessions + 4 telephone calls for continuous reinforcement (4*0.5 = 2 sessions)<br>6 / 22 = 27.3%<br>**Duration:** ≥ 12-months<br>**Fidelity:** Not clear |
| Janus et. al. 2012 [46] | pMDPS | Low | Moderate | High | **Frequency:** The intervention was a series of six structured 90-minute group sessions, five fortnightly sessions and the final session at 8 months after the first.<br>6/22= 27.3%<br>**Duration:** 8 months<br>**Fidelity:** This evaluation is being undertaken to see the effect of scaling-up to state level from the small GGT DPP implementation trial.<br>The Finnish Diabetes Prevention Study goals were used. |

| Implementation | | | | | |
|---|---|---|---|---|---|
| **Author Year** | **Study ID** | **Frequency** | **Duration** | **Fidelity** | **Description** |
| Kanaya et. al. 2012 [50] | LWBW | Moderate | Moderate | Moderate | **Frequency:** It was delivered in Spanish and English and consisted of a 6-month active intervention phase and a 6-month maintenance phase. 1 introductory session; 1 in-person planning session; 12 telephone counseling calls.<br>1 + 1 + 6 (12 telephone calls * 0.5) = 8/22 = 36%<br>**Duration:** 12 months<br>**Fidelity:** Not clear in the article, however, the project's internet site mentions about a standard curriculum consisting of materials for program users and participants. |
| Lakerveld et. al. 2012 [37] | Hoorn Prevention Study | Moderate | Moderate | High | **Frequency:** Six individual 30-minute counselling sessions, followed by 3-monthly booster sessions by phone for a period of one year.<br>6 + 1.5 (3 telephone sessions * 0.5) = 7.5/22 = 34.09%<br>**Duration:** 12 months<br>**Fidelity:** We assessed the extent to which the various intervention components were delivered as intended. The counselling sessions were tape-recorded to allow assessment of the validity of MI and PST provided by the practice nurses. Two tape-recorded sessions of ≥ 15 minutes from all practice nurses were drawn at random using a computerized randomization program and transcripts were made; all final samples were then independently analyzed by two researchers. Because not all samples contained sessions in which PST was used, 10 extra random sessions have been independently analyzed using the PSCC. |
| Ockene et. al. 2012 [80] | LLDPP | High | Moderate | Moderate | **Frequency:** Three individual and 13 group sessions over a 12-month period. 16/22=72.7%<br>**Duration:** 12 months<br>**Fidelity:** Intervention fidelity was facilitated through extensive training in the delivery of the intervention protocol, including the nutritional & exercise aspects of the intervention, the theoretical background, and training in motivational counselling and group management skills. |
| Piatt et. al. 2012 [81] | GLB (2005-2008) | Moderate | Moderate | Moderate | **Frequency:** Twelve weekly sessions over 12-14 weeks (lasted ~90 min) in the groups of 5 to 13 participants. 12/22=54.5%<br>**Duration:** 12 months<br>**Fidelity:** The DPP lifestyle intervention was adapted to a 12-session group-based program. |
| Jiang et. al. 2013 [82] | SDPI-DP | High | Moderate | Moderate | **Frequency:** The curriculum was delivered in group settings within 16-24 weeks after baseline assessment and typically was taught by the program dietitian and/or health educator. It was supplemented by monthly individual lifestyle coaching sessions to individualize goals and plan and to identify and solve barriers to participation. 22/22=100%<br>**Duration:** 12 months<br>**Fidelity:** Based on the DPP lifestyle intervention. |

| Implementation | | | | | |
|---|---|---|---|---|---|
| **Author Year** | **Study ID** | **Frequency** | **Duration** | **Fidelity** | **Description** |
| Ma J et. al. 2013 [38] | E-LITE | High | High | High | **Frequency:** E-LITE was a 3-arm, primary care–based randomized trial designed to evaluate the effectiveness of 2 adapted DPP lifestyle interventions: (1) a coach-led, face-to-face group intervention and (2) a self-directed DVD intervention. Participants in both intervention groups completed a 3-month intensive intervention phase and a 12-month maintenance phase. During the intensive intervention phase, participants received an adapted, 12-session DPP lifestyle intervention curriculum. The curriculum was delivered face-to-face in 12-weekly classes to coach-led intervention participants or via a home-based DVD to self-directed intervention participants. Coach-led intervention participants had food tastings at check-in and 30 to 45 minutes of guided physical activity at the end of each weekly class.<br>[Twelve weekly group sessions (1.5 - 2hrs each) in the first 3 months. From month 4–15, contact every 2-4 week depending on participant needs and preferences. Individual, secure email/phone contacts with personalized progress feedback and lifestyle coaching through the maintenance phase (mo4-15).]<br>**Duration:** $\geq$ 12 months<br>**Fidelity:** To assure intervention fidelity, the DPSC provides training and certification of instructors who will be delivering the intervention to patients. The E-LITE dietitian lifestyle coach completed this DPSC instructor certification. During group sessions, the DPSC group-based DPP intervention manual was followed, with the only exceptions being two additional activities added to all group sessions: a food-tasting at check-in and a 30- to 45-min physical activity demonstration and practice at the session end. |
| Duijzer et. al. 2014 [49] | SLIMMER | Low | Moderate | Moderate | **Frequency:** A dietician gave tailored dietary advice during six individual consultations within the 10-month intervention period (30–60 min per consultation; in total 4 h per participant). In addition, the dietician organised one group session.<br>The physical activity intervention consisted of a combined aerobic and resistance exercise programme (proportion 2:1) at the physiotherapist's practice. Weekly training sessions with a duration of 1 h were group-based and supervised by a skilled physiotherapist. Subjects had free access to the training sessions and were stimulated to participate for at least 1 h per week.<br>6 + 1 session = 7/22 = 31.8% Low<br>**Duration:** 10 months<br>**Fidelity:** The SLIMMER intervention resembles the SLIM intervention, which was based on the Finnish Diabetes Prevention Study.<br>Overall, the intervention was implemented as planned. Some parts of the protocol, however, were omitted or adjusted by health care professionals. This mostly concerned measurements and planning aspects regarding intervention elements. |

| Implementation | | | | | |
|---|---|---|---|---|---|
| **Author Year** | **Study ID** | **Frequency** | **Duration** | **Fidelity** | **Description** |
| Sepah et. al. 2014 [47] | Prevent | Moderate | High | Moderate | **Frequency:** The DPP curriculum was presented in an asynchronous online format that resembled popular online learning platforms such as Coursera. The Prevent program began with a 16-week core program phase, consisting of 16 online weekly lessons adapted from the CDC National DPP core curriculum.<br>Once participants completed the 16-week core phase, they were invited to participate in the post-core phase, totalling 12 months. The post-core phase included 9 monthly lessons from the CDC National DPP post-core curriculum. The post-core phase differed in that all groups were combined into a larger participant-led “super-group” and focused on maintaining lifestyle habits and weight loss achieved during the core program.<br>16 + 9 = 25 (online sessions = 25 * 0.5 sessions = 12.5 sessions)<br>12.5/22 = 56.8%<br>**Duration:** $\geq$ 12 months<br>**Fidelity:** The Prevent program was designed to provide delivery of the DPP lifestyle intervention in an online small group format that is accessible and engaging. Prevent included 4 major intervention components: small-group support, health coaching, DPP curriculum, and digital tracking tools.<br>Each group was led by a professional health coach, who was trained in a manner consistent with CDC DPRP standards for lifestyle coaches. |
| Zyriax et. al. 2014 [34] | DELIGHT | High | High | Low | **Frequency:** Twelve weekly sessions (for the first 6 months), six monthly and six biweekly sessions (for the next 6mo). For year 2 & 3, quarterly 1.5-h sessions. 18/22=81.8%<br>**Duration:** > 12-months<br>**Fidelity:** Initially, 6 sessions of dietary advice according to the Finnish DPS, changed every other week with 6 sessions on advice and motivation for physical activity including a walking program. |
| Savas et. al. 2015 [44] | IGT Care Call | Low | Moderate | High | **Frequency:** 6 months of telephone calls.<br>Introduction call (10 min); action planning call (40 min); and monthly call (20 min * 6) 8 calls = 4 sessions i.e. 4/22 = 18%<br>**Duration:** 6 months<br>**Fidelity:** Based on the article where the intervention is described and the figure describing the calls, fidelity if being rated “high”. |

## Supplementary Table 6 – Scoring of all included studies (PARTICIPATION)

| Participation | | | |
|---|---|---|---|
| **Author Year** | **Study ID** | **Scoring** | **Description** |
| Mensink et. al. 2003 [35] | SLIM | Low | Invited (6,108), 1st OGTT done (2,820), eligible (379), 2nd OGTT done (177), enrolled (114) [114/6,108 = 0.0186 (1.9%)] |
| Kosaka et. al. 2005 [62] | Japanese DPP | NAC | One of every five subjects was randomly selected for allocating to the intervention group, and the others were assigned to the control group. After 1 year of observation, the number of subjects in the control group and the intensive intervention group was 356 and 102 respectively. |
| Oldroyd et. al. 2006 [63] | Newcastle Lifestyle Intervention | Low | Identified with IGT (498), excluded (51), declined/NR (239), re-tested (208), excluded (126), recruited/randomised (78) [78 / 498 = 0.156 (15.6%)] |
| Absetz et. al. 2007 [41] | GOAL LIT | High | Risk status was screened in 462 patients. After exclusions, 352 were eligible and enrolled. [352 / 462 = 0.76 (76%)] |
| Bo et. al. 2007 [36] | Italian Trial | Low | Individuals contacted (1,877), total screened for MS (1,658), metabolic abnormalities present (503), excluded for ineligibility (128), randomized (375), refused to participate (40), total enrolled (335) [335 / 1877 = 0.178 (17.8%)] |
| Davis-Smith et. al. 2007 [39] | DPP (church-based) | Low | Invited for risk assessment (150), enrolled (10) [10 / 150 = 0.066 (6.7%)] |
| Laatikainen et. al. 2007 [64] | Greater Green Triangle (GGT) | Low | Participants approached (>1500), total participated (311) [311 / 1500 = 0.207 (20.7%)] |
| Ackermann et. al. 2008 [65] | DEPLOY | Low | Adults assessed (n = 535), 131 were eligible. 39 were not interested, and 92 were enrolled. [92 / 535 = 0.17 (17%)] |
| Boltri et. al. 2008 [66] | DPP (church-based | Low | Risk assessment completed (50), high-risk identified (26), excluded (4), refused (6), screened for FGT (16), eligible (8), and enrolled (8). [8/50 = 0.16 (16%)] |
| Payne et. al. 2008 [67] | BDPPI | NAC | A total of 122 adults were recruited from the city of Ballarat. |
| Kramer et. al. 2009 [68] | GLB (2007 – 2009) | Low | Patient base (15,000), referrals received (74), eligible (56), enrolled (42). [42 / 15000 = 0.0028 (0.28%)] |
| Kulzer et. al. 2009 [69] | PREDIAS | NAC | Participants randomized (182) |
| Penn et. al. 2009 [70] | EDIPS- Newcastle | Low | Patients contacted (1,567), patient replied (1,084), agreed to testing (682), completed 1st OGTT (482), eligible (153), completed 2nd OGTT (141), recruited (102). [102 / 1567 = 0.065 (6.50%)] |
| Almeida et. al. 2010 [71] | Colorado Weight Loss Intervention | Low | Of the 12,468 eligible participants, 1,030 participated. [1030 / 12468 = 0.0826 (8.26%)] |
| Makrilakis et. al. 2010 [72] | DE-PLAN Greece | Low | FINDRISC distribution (7,900), completed ques. received (3,240), high-risk identified (620), accepted an OGTT (318), high-risk identified (251), enrolled (191) [191 / 7900 = 0.024 (2.4%)] |

| **Participation** | | | |
|---|---|---|---|
| **Author Year** | **Study ID** | **Scoring** | **Description** |
| Parikh et. al. 2010 [73] | Project HEED | Low | Over 3 months, 555 people were approached and screened. The consent was obtained from 249. Oral glucose tolerance tests was performed on 178 and 99 were randomised. [99 / 555 = 0.178 (17.8%)]. |
| Vanderwood et. al. 2010 [45] | Montana CDDP | High | Target pop. (n = 400). Participants enrolled 355. [355 / 400 = 0.887 (88.7%)] |
| Vermunt et. al. 2010 [74] | APHRODITE | Low | FINDRISC questionnaire was sent to (16,032), FINDRISC ques. returned (8,752), high-risk patients identified (1,533), interviews (1,065), OGTT (1,024), participation (925) [925 / 16,032 = 0.057 (5.77%)] |
| Boltri et. al. 2011 [75] | DPP (church-based) | Low | Risk test done on (442), identified as high-risk (234), went for further screening (180), identified as high-risk (74), and enrolled (37). [37 / 442 = 0.0837 (8.4%)] |
| Gilis-Januszewska et. al. 2011 [76] | DE-PLAN Poland | Low | FRS questionnaire distributed (800), questionnaire completed (566), high-risk identified (368), agreed for OGTT (275), enrolled (175). [175 / 800 = 0.2187 (21.9%)] |
| Katula et. al. 2011 [77] | HELP PD | Low | Of the 1,818 initial telephone screening completed, 301 participants (16.6%) were eventually randomized. |
| Kumanyika et. al. 2011 [48] | Think Health! | Moderate | Pre-screened (412), not eligible (52), assessed for further eligibility (284), not eligible (19), randomized (261) [261 / 412 = 0.633 (63.3%)] |
| Nilsen et. al. 2011 [42] | Nilsen | High | Individuals referred (234), randomised (213) [(213 / 234 = 0.91 (91%)) |
| Penn et. al. 2011 [43] | NLNY | High | Register of interest (271), excluded (33), assessment (238), excluded (21), recruited (217) [217 / 271 = 0.80 (80%)] |
| Ruggiero et. al. 2011 [78] | HLP | Low | Individuals assessed (1,162), screened for eligibility (367), eligible to participate (244), interested in participating (120), enrolled (69) [69/1162 = 0.059 (5.93%)] |
| Sakane et. al. 2011 [79] | Japanese Study | Low | Pre-screened (1,279), excluded (975), randomized (304) [304 / 1279 = 0.237 (23.7%)] |
| Costa et. al. 2012 [40] | DE-PLAN-CAT | Low | Invited (2,547), screened (2,054), received an OGTT (1,192), high-risk (624), agreed to participate (552) [552 / 2547 = 0.2167 (21.67%)] |
| Janus et. al. 2012 [46] | pMDPS | High | Individuals recruited (99), participants excluded (7), participants enrolled (92). [92 / 99 = 0.929 (93%)] |
| Kanaya et. al. 2012 [50] | LWBW | Moderate | Assessed for study eligibility (544), excluded (117), refused (189), randomized (238) [238 / 544 = 0.4375 (43.7%)] |
| Lakerveld et. al. 2012 [37] | Hoorn Prevention Study | Low | Mail invitation sent (8,193), individuals responded (3,587), eligible (772), and randomised (622) [622 / 8193 = 0.0759 (7.59%)] |

| Participation | | | |
|---|---|---|---|
| **Author Year** | **Study ID** | **Scoring** | **Description** |
| Ockene et. al. 2012 [80] | LLDPP | Low | Telephone screening calls (9,959), screening completed (2,638), 2nd screening scheduled (1,296), screening conducted (949), eligible (391), recruited (312) [312 / 9,959 = 0.031 (3.1%)] |
| Piatt et. al. 2012 [81] | GLB (2005-2008) | Low | Total attended screening (638), eligible (203), enrolled (105) [105/638 = 0.164 (16.4%)] |
| Jiang et. al. 2013 [82] | SDPI-DP | Low | Individuals identified for screening (18,134), participants enrolled (2,615), started intervention (2,553) [2615 / 18134 = 0.144 (14.4%)] |
| Ma J et. al. 2013 [38] | E-LITE | Low | Contact attempted for screening (2,391), randomized (241) [241 / 2391 = 0.100 (10.0%)] |
| Duijzer et. al. 2014 [49] | SLIMMER | Moderate | Invitation letter sent (54), no response (20), willing to participate (34), excluded (3), enrolled (31) [31 / 54 = 0.574 (57.4%)] |
| Sepah et. al. 2014 [47] | Prevent | High | Identified for screening and recruitment (254), started intervention (220) [220 / 254 = 0.866 (86.6%)] |
| Zyriax et. al. 2014 [34] | DELIGHT | Low | Participants invited (3,000), enrolled (241) [241 / 3000 = 0.080 (8.0%)] |
| Savas et. al. 2015 [44] | IGT Care Call | High | 55 people were referred to IGT Care Call. All were invited and agreed to participate in the service and received 6 months of telephone call. [55 / 55 = 1 (100%)] |

## Supplementary Table 7 – Scoring of all included studies (EFFECTIVENESS)

| EFFECTIVENES | | | | | | |
|---|---|---|---|---|---|---|
| **Author Year** | **Study ID** | **Success Rate** | **Weight Loss** | **Risk Reduction** | **Description** | **Intention-to-treat** |
| Mensink et. al. 2003 [35] | SLIM | NAC | Moderate | High | Moderate weight loss mean intervention –2.77 kg vs. control –0.62kg, but high relative risk reduction in intervention 48%. | - |
| Kosaka et. al. 2005 [62] | Japanese DPP | NAC | Moderate | High | Mean weight decreased by 2.5 kg after 1-year and tended to increase slightly thereafter, but remained significantly lower (by 2.18 kg) than the baseline value at the end of 4 years.<br>The cumulative incidence of diabetes in the intervention group during the 4yrs was 3.0%, which was significantly less than the control group (9.3%). Thus the development of diabetes had been reduced by 67.4% in the intervention group. | - |
| Oldroyd et. al. 2006 [63] | Newcastle Lifestyle Intervention | NAC | Low | NR | Weight loss after 6 months was -1.1 kg in intervention and +0.54kg in control group, and after 24-months was -1.8 kg vs. +1.5 kg in control group. | All statistical analyses, were performed on an intention-to-treat basis. |
| Absetz et. al. 2007 [41] | GOAL LIT | Low | Low | NR | Small effect in average weight loss at 12-month, Females 0.5 kg, Males 1.5 kg. The 5% weight loss objective was significantly less frequently achieved in the trial. < 20% achieved the 5% weight loss goal. | - |

| EFFECTIVENES | | | | | | |
|---|---|---|---|---|---|---|
| **Author Year** | **Study ID** | **Success Rate** | **Weight Loss** | **Risk Reduction** | **Description** | **Intention-to-treat** |
| Bo et. al. 2007 [36] | Italian Trial | NAC | Low | High | Weight loss: -0.75kg in the intervention group vs. 1.63kg in controls. The intervention group significantly reduced MS with a 31% absolute risk reduction. Overall, after a 1-year follow-up, the cumulative incidence of diabetes was 4.5% (n = 15). Even if the number of events was small, the lifestyle intervention significantly reduced progression to diabetes (1.8% vs. 7.2%). Incidence reduction 75% | The exclusion of the subjects, who refused to participate, did not seem to affect results because they were well-balanced between both groups. Moreover, the sensitivity analysis based on the intent-to-treat population confirmed the results based on participants only. |
| Davis-Smith et. al. 2007 [39] | DPP (church-based) | NAC | High | NR | Mean weight loss for the combined group when compared to the initial weight was -10.6 lbs at 12 months = 4.8 kg | A pre-to-post comparison was made for each subject using an intention-to-treat analysis. |
| Laatikainen et. al. 2007 [64] | Greater Green Triangle (GGT) | NAC | Moderate | NR | At 12-months participants' mean weight reduced by 2.52kg (2.7%) | - |
| Ackermann et. al. 2008 [65] | DEPLOY | NAC | High | NR | At 12-months weight decreased by 6.0% (intervention) and 1.8% in controls. Weight loss of 5.7 kg (Intervention) vs. 1.6 kg for controls. | - |
| Boltri et. al. 2008 [66] | DPP (church-based | NAC | Low | NR | From baseline to 12-mo, the change in weight was -1.0 lbs. = 0.45 kg | We performed a pre-post comparison for each participant using SPSS and an intention to treat analysis. |
| Payne et. al. 2008 [67] | BDPPI | Moderate | Moderate | NR | Mean weight loss was -4.07kg. 39.3% participants decreased weight by at least 5% at 12-months. | The basis of the analysis was intention to treat. The designated post-intervention data collection point was week 52. In individuals for whom no week 52 data were available (lost to follow-up), the last available data were carried forward. |
| Kramer et. al. 2009 [68] | GLB (2007 – 2009) | NAC | High | NR | Weight loss per protocol was significant with an average decrease of 11 lbs (-5.3%) at 12-months. = 4.99 kg | Measures were made of change in risk parameters for subjects in both urban and rural environments in two phases, with data analyzed according to the intention-to-treat principle as well as per protocol. |

| EFFECTIVENES | | | | | | |
|---|---|---|---|---|---|---|
| **Author Year** | **Study ID** | **Success Rate** | **Weight Loss** | **Risk Reduction** | **Description** | **Intention-to-treat** |
| Kulzer et. al. 2009 [69] | PREDIAS | NAC | Moderate | NR | After 12 months, weight loss was significantly higher in PREDIAS (-3.8kg) (4.1%) than in the control group (-1.4kg). | An intention-to-treat analysis was performed using the baseline observation carried forward method. Statistical analyses were performed by paired *t* tests for within-group differences and independent *t* tests for between-group differences |
| Penn et. al. 2009 [70] | EDIPS-Newcastle | NAC | Low | High | At 12-months, mean weight change in intervention group was -2.3 kg vs. 0.01 kg in the control group.<br>The absolute incidence of T2D was 32.7 per 1000 person-years in the intervention group and 67.1 in the control group. The overall incidence of diabetes was reduced by 55% in the intervention group. | - |
| Almeida et. al. 2010 [71] | Colorado Weight Loss Intervention | Low | Low | NR | Weight at 12-months decreased significantly more than that of their matched controls (mean WL -3.0 lbs vs. -1.4). =1.4 kg<br>A significantly higher proportion of participants lost at least 5% weight and were 1.5 times more likely to lose at least 5% of their body weight that their matched controls.<br>1 in 5 (i.e. 20%) participants lost atleast 5% of their initial body weight. | - |
| Makrilakis et. al. 2010 [72] | DE-PLAN Greece | NAC | Low | NR | Average weight loss was 1.0 kg. Weight loss was significant only in participants who completed 4–6 intervention sessions (1.1+ 4.8 kg) i.e. about 58% of total participants. | - |

| EFFECTIVENES | | | | | | |
|---|---|---|---|---|---|---|
| **Author Year** | **Study ID** | **Success Rate** | **Weight Loss** | **Risk Reduction** | **Description** | **Intention-to-treat** |
| Parikh et. al. 2010 [73] | Project HEED | Moderate | Moderate | NR | Sixteen intervention participants (34%) lost at least 5% of their baseline weight in 12 months; only 6 control participants (14%) achieved this. At 12 months, intervention participants had lost on average 7.2 pounds, or 4.3% of their baseline weight. = 3.3 kg | In this intention-to-treat analysis with weight as our primary outcome, we used a last-observation-carry-forward strategy to impute missing weights at follow-up. |
| Vanderwood et. al. 2010 [45] | Montana CDDP | High | High | NR | Among the after core completers, 49% met the 7% weight loss, and 64% achieved 5% weight loss. WL at 12-mo: 7.7kg. | - |
| Vermunt et. al. 2010 [74] | APHRODITE | NAC | NAC | NR | Both groups showed small but significant reductions in BMI, however, that was less pronounced after 1.5 years. Weight loss not reported in the primary analysis. | - |
| Boltri et. al. 2011 [75] | DPP (church-based) | NAC | Low | NR | While weight decreased 1.7 kg after the intervention, by the 12-month follow-up, there was an average of 0.9 kg regained. | Analyses were based on an-intent-to-treat basis. For missing values, the most conservative value was used, eg, the highest weight was substituted for a missing weight. |
| Gilis-Januszewska et. al. 2011 [76] | DE-PLAN Poland | Low | Low | NR | At 12-mo, mean weight reduced by 1.9 ± 5.0 kg (2.2%). 24.6% of study participants lost 5% or more of their initial body weight, 40% lost less than 5% of initial body weight. 14.7% did not change their weight and 21.7% gained weight. | - |
| Katula et. al. 2011 [77] | HELP PD | NAC | High | NR | Participants in the intervention group lost an average of -7.1kg (7.3%) at 12 months. At 12-mo, intervention participants lost 6% more weight than control group. | We used the intention-to-treat approach and included all post-randomized values according to the group to which they were assigned. We performed secondary analyses, making a reasonable exception to this rule by deleting observations at visits where the subject was taking hypoglycemic medication. |

| EFFECTIVENES | | | | | | |
|---|---|---|---|---|---|---|
| **Author Year** | **Study ID** | **Success Rate** | **Weight Loss** | **Risk Reduction** | **Description** | **Intention-to-treat** |
| Kumanyika et. al. 2011 [48] | Think Health! | Low | Low | NR | 1-year weight change were -1.61kg in Basic Plus and -0.62kg in Basic group (difference 0.98).<br>More Basic Plus (22.5%) than Basic (10.2%) participants lost >5% of their baseline weight. | The actual date the measurement was taken was included to permit treatment group contrasts based on estimated weight at 0 and 12 months. Participant level analyses were "as randomized" (intention to treat) |
| Nilsen et. al. 2011 [42] | Nilsen et. al. | Moderate | NAC | NR | At least 5% weight loss was achieved by 35% (IG) and 28% (IIG). | - |
| Penn et. al. 2011 [43] | NLNY | Low | Low | NR | Mean change in weight: (in men) from baseline to 6-mo: -4.2kg, from 6- to 12-mo: -1.5kg; and (in women) from baseline to 6-mo: -2.2kg, from 6- to 12-mo: -0.6kg.<br>About 21% of participants lost ≥5% of their body weight at 12 months of follow-up. | - |
| Ruggiero et. al. 2011 [78] | HLP | Moderate | Low | NR | At 12-mo, (30% achieved a 5% goal). The average WL at 12-months was -2.79 lbs (1.3kg). | - |
| Sakane et. al. 2011 [79] | Japanese Study | NAC | Low | High | After 1 year, the intervention group had very modest but significant improvement in body weight: -1.5 ± 2.7kg (2.3%) vs. -0.7 ± 2.5kg (1.3%) in the control group. The 3-year cumulative incidence tended to be lower in the intervention group (14.8% vs. 8.2%). The RR reduction was thus 53% with the intervention. | - |

| EFFECTIVENES | | | | | | |
|---|---|---|---|---|---|---|
| **Author Year** | **Study ID** | **Success Rate** | **Weight Loss** | **Risk Reduction** | **Description** | **Intention-to-treat** |
| Costa et. al. 2012 [40] | DE-PLAN-CAT | NAC | Low | High | LOW weight loss but MODERATE effect on 4 year incidence.<br>24.7% in intervention vs. 18.8% in control reduced weight by at least 3%, The absolute incidences of diabetes were 7.2 cases per 100 person-years in standard care and 4.6 in intervention ($p<0.005$, logrank test, and risk reduction in intervention group 36%. | Analyses after 4-year follow-up were performed based on the intention-to-treat principle with comparison of standard care and the combined intervention groups.<br>Finally, diabetes diagnoses of all individuals who discontinued the protocol were ascertained and included in the intention-to-treat analysis.<br>All comparisons were based on the intention-to-treat principle.<br>..we included 19 (8.4%) diabetes-related diagnoses (11 individuals allocated into the standard care group and eight into the intensive group) in the intention-to-treat analysis. |
| Janus et. al. 2012 [46] | pMDPS | NAC | Moderate | Moderate | At least 5% weight loss was achieved by 32% of the intervention group compared with none of the controls. Participants achieved 24% diabetes risk reduction based on weight reduction. | Differences between groups for changes over 12 months were originally analysed using analysis of covariance, adjusted for baseline values. Subsequently, generalized estimating equations were used to satisfy the intention-to-treat principle, including 3-month data (collected for intervention only) in the model. |
| Kanaya et. al. 2012 [50] | LWBW | NAC | Low | NR | At 12-mo, weigh reduced -1.34 lbs (0.6 kg) in intervention group and -0.42 lbs (or 0.2 kg) in the control group. The intervention group had significantly more weight loss than did the control group. | Intention-to-treat linear regressions modeled repeated change scores: outcomes assessed at each follow-up (6 or 12 months) minus the corresponding baseline value. |
| Lakerveld et. al. 2012 [37] | Hoorn Prevention Study | NAC | NAC | NR | "There were NO or very small differences in determinants of lifestyle behavior between groups at both follow-ups." | The analyses were all based on the intention-to-treat principle. |

| EFFECTIVENES | | | | | | |
|---|---|---|---|---|---|---|
| **Author Year** | **Study ID** | **Success Rate** | **Weight Loss** | **Risk Reduction** | **Description** | **Intention-to-treat** |
| Ockene et. al. 2012 [80] | LLDPP | NAC | Low | NR | Compared with the usual care group, the intervention group had a modest but significant weight reduction (-2.5 lbs vs. 0.63lbs). = 1.1. kg | - |
| Piatt et. al. 2012 [81] | GLB (2005-2008) | Moderate | NAC | NR | Of the participants who lost at least 5% of their body weight at 3 months, 52.6% maintained the 5% weight loss at their last follow-up time (24 months). The probability of being at risk for diabetes and CVD was significantly reduced by 25.7% over the 24-months. | - |
| Jiang et. al. 2013 [82] | SDPI-DP | NAC | Moderate | NR | Participants had significant weight loss at each clinical assessment after baseline. The average weight loss attenuated over the three annual visits to 5.6lbs (2.5 kg), 3.1lbs and 2.4lbs respectively. The crude incidence of diabetes was 4% per year. | - |
| Ma J et. al. 2013 [38] | E-LITE | High | High | NR | At month 15, the mean change in weight from baseline was -6.3 kg in the intervention and -2.4kg in the control group, corresponding to a weight change of -6.6%, and -2.6% respectively. Intervention group sustained significantly greater weight loss at 15-mo compared to controls, with approx. 50% achieving 5% or greater WL from baseline. | Between-group differences in primary and secondary outcomes were evaluated by intention-to-treat using tests of group by time interactions in repeated-measures mixed-effects linear (for continuous outcomes) or logistic models (for categorical outcomes). |
| Duijzer et. al. 2014 [49] | SLIMMER | NAC | Moderate | NR | Participants' weight loss was -3.5 kg. | Quantitative data analyses are performed following the intention-to-treat procedure. |
| Sepah et. al. 2014 [47] | Prevent | High | NAC | NR | In the post-core participants who reported weight between 11 and 13 months (N = 135), 47% met or exceeded the 5% weight loss benchmark at 12 months. | - |
| Zyriax et. al. 2014 [34] | DELIGHT | NAC | NAC | NR | At 12-mo, only 26.1% of participants lost weight, while all others showed no change and the majority had even gained weight. | - |

| EFFECTIVENES | | | | | | |
|---|---|---|---|---|---|---|
| **Author Year** | **Study ID** | **Success Rate** | **Weight Loss** | **Risk Reduction** | **Description** | **Intention-to-treat** |
| Savas et. al. 2015 [44] | IGT Care Call | NAC | Moderate | NR | The majority of participants (88%) achieved or partially achieved their 6 month lifestyle goal. At 18-mo, in comparison with baseline, these positive changes were sustained, with reductions of 2.81 kg in weight and 1.06kg/m2 in BMI. | - |

*Note: RR = Risk Reduction; WL = Weight Loss*
